# Analysis of bottleneck technology identification and development characteristics in the electronic manufacturing industry

**DOI:** 10.1371/journal.pone.0310176

**Published:** 2024-12-19

**Authors:** Lei Zhou

**Affiliations:** Beijing Institute of Science and Technology Information, Beijing Academy of Science and Technology, Beijing, China; Universiti Teknologi Malaysia, MALAYSIA

## Abstract

In 2018, US government imposed a ban on Chinese electronics firm ZTE, prohibiting it from procuring chip products from American companies for a duration of seven years. Subsequently, ZTE experienced a shutdown. The concept of "bottleneck technology" gained prominence in diverse media outlets. Chinese government acknowledged the importance of identifying pertinent bottleneck technologies in specific sectors, particularly in electronic manufacturing. It underscored the significance of promptly analyzing the evolutionary traits of these technologies within the industry and engaging in research and development endeavors to safeguard national technological security. To address this issue, the study initially extracted pertinent industry enterprises from the Orbis database. Subsequently, it utilized the technical codes associated with the patent data of these enterprises to establish a social network, pinpoint key technologies, and compute the technical share metrics for these pivotal technology codes. Numerous technologies are exclusively controlled by a limited number of enterprises, leading to the identification of bottleneck technologies. This, in turn, enables the determination of critical paths to reveal the developmental history and distinctive features of these bottlenecks. The research indicates that the electronic manufacturing industry faces various bottleneck technologies, which extend beyond chips to include production equipment, materials, processes, and inspection devices. Numerous technologies have a significant developmental background, necessitating substantial technological development to achieve parity. However, in swiftly advancing technological domains, China possesses the capability to make advancements at an accelerated rate.

## 1. Introduction

In 2018, U.S.-China trade friction erupted. U.S., citing the trade deficit with China, imposed tariffs on Chinese exports while also restricting imports from China and implementing technology knowledge barriers. The "ZTE incident", in particular, served as a direct catalyst for the focus on bottleneck technologies. In the same year, U.S. Department of Commerce issued an announcement stating that U.S. government would prohibit ZTE Corporation from purchasing sensitive products from U.S. enterprises for the next seven years. These sensitive products encompassed components, commodities, software, and technology, including core components of ZTE products, such as semiconductor chips. Prior to this action, ZTE relied on imports from U.S. for more than half of its semiconductor chips. As a result, ZTE’s stocks were suspended both in the A-share and H-share markets, and the enterprise faced a state of "shock". Following negotiations and mediation, ZTE ultimately agreed to a significant penalty of $1 billion and a substantial restructuring of its board of directors in exchange for the lifting of the ban. The "ZTE incident" highlights the critical role that bottleneck technologies play in the normal course of an enterprise’s production and business operations. When an enterprise cannot independently develop such technology and lacks alternative products, relying solely on one or two suppliers or source countries, any disruption in the supply chain or export restrictions by the supplier or source country can inflict a devastating blow on the enterprise, posing a threat to national economic security. Subsequently, the term bottleneck technology has increasingly appeared in various Chinese media outlets and research discussions. Meanwhile, the timely identification of bottleneck technologies and the implementation of research and development efforts to address them have emerged as pressing priorities for Chinese governments at various administrative tiers.

Bottleneck technology has garnered significant attention at the research level. The primary focus of the initial research was the identification of bottleneck technologies to address potential issues proactively. Tang et al. (2020) employed a questionnaire survey to identify 13 bottleneck technologies in the electronic information industry [1]. Li (2021) analyzed industry reports from diverse sources and examined the core issues related to innovation in the seed industry within the context of international competition [2]. This approach allowed for the identification of key bottleneck technologies in the seed industry competition. Guang et al. (2021) focused on bottleneck technologies in China’s oil and gas engineering sector, which encompassed fine exploration technology, deep-sea oil and gas engineering technology, ultra-high-temperature downhole tools, high-performance intelligent drilling guidance technology, oil and gas reservoir precision redevelopment technology, and integrated decision-making technology in geological engineering [3]. Liang et al. (2021) analyzed bottleneck technologies in the high-end Computer Numerical Control (CNC) machine tool industry by examining the number of patents [4]. Zheng et al. (2021) utilized Delphi method and Analytic Hierarchy Process to select bottleneck technologies in the field of biomedicine [5].

Simultaneously, the strategies to overcome and surpass bottleneck technologies have emerged as another focal point of research. Chen et al. (2020) propose that the decryption of bottleneck technologies necessitates strengthening the development of a science and technology security early warning and monitoring system [6]. Moreover, it emphasizes the importance of enhancing collaboration between enterprises and research institutions. Depending on the distinctive characteristics of different industries and types of innovation, it is essential to leverage the roles of various innovation entities to break through these bottleneck technologies. Similarly, Tang et al. (2020) argue that addressing bottleneck technologies in the information industry entails a dual-pronged approach [1]. On the technological front, there is a need to elevate the level of basic research. Simultaneously, from an industrial perspective, the strategy should commence at the source, focusing on upstream development within the industry chain and positioning in the high-value segments of the value chain. This approach should fully harness the advantages of the socialist system, with a particular emphasis on concentrating resources internally to achieve breakthroughs in "bottleneck" technologies within the industry. Furthermore, Jing et al. (2021), when discussing bottleneck technologies in the context of agricultural seed resources, advocate for the advancement of reforms in state-owned research institutions [7]. This transformation aims to fundamentally change the prevailing dual-track system in China’s crop breeding research. They also recommend the establishment of a policy support system for the crop seed industry, with enterprises taking the lead. This approach is seen as instrumental in fostering the healthy development of domestic seed enterprises.

From the aforementioned research, several key observations can be made. Firstly, current research predominantly relies on methods such as questionnaire surveys, the number of relevant patents within the industry, industry reports, and analytic hierarchy processes for the identification of bottleneck technologies. However, there is no universally accepted or standardized method for this purpose. Importantly, the identification and screening of bottleneck technologies serve as early warning mechanisms. Hence, automating the identification of bottleneck technologies through a series of readily available market data sources, complemented by the analysis of interconnected information, is advantageous. Secondly, regarding the challenge of breaking through and surpassing bottleneck technologies, existing research primarily originates from a policy perspective. It suggests that various innovation entities should collaborate through joint efforts, leveraging the national system and harnessing the vast Chinese market to expedite the development of domestic products and mitigate the impact of bottleneck technologies. However, there is a limited discussion on the technological development and diffusion aspects. As previously mentioned, bottleneck technologies are conventional technologies with a significant history of development. Consequently, analyzing the knowledge diffusion and evolutionary characteristics required for the development of such technologies is imperative. Such analysis can guide and accelerate breakthroughs in bottleneck technologies across different industries. This paper firstly elucidates a method for identifying bottleneck technologies using a electronic manufacturing industry case study, utilizing commercial databases. Subsequently, an analysis and discussion of the technological diffusion and evolutionary characteristics of the identified bottleneck technologies are presented. After acquiring these bottleneck technologies, the crucial next step in the research process is to uncover the technological development characteristics associated with these bottleneck technologies. In the future, should China decide to disengage from these technologies, it will be essential for the country to familiarize itself with the developmental trajectory and distinctive features of these technologies to facilitate its efforts to catch up. Furthermore, an examination of the evolution of these bottleneck technologies can significantly assist Chinese scientific research management departments in devising development strategies for analogous technologies and expediting the research and development endeavors related to these technologies. Finally, the characteristics of the evolution of these bottleneck technologies are summarized, and insights into future trends are provided, along with recommendations for China to overcome these technologies in electronic manufacturing.

This primarily involves two issues that have not been addressed in previous research, which are outlined as follows:

(1) What is the specific stage within an industry chain where bottleneck technologies are typically located, and how can these bottleneck technologies be effectively identified?

Based on previous research, bottleneck technologies emerge within the context of an industry chain. Monopolistic technologies at different stages of the industry chain constitute bottleneck technologies. Since an industry chain encompasses all processing and production stages from raw materials to finished products, including the equipment and auxiliary materials used for processing and production, it consists of enterprises functioning at various stages. Therefore, when conducting patent analysis using the industry chain as a framework, it is crucial to address the following questions: the composition of enterprises involved in each stage of the industry chain, the technological advantages of these enterprises at each stage, and the presence of upstream and downstream constraints. Obtaining this information can often be challenging and time-consuming.

Traditionally, patent analysis can shed light on an enterprise’s strengths and weaknesses in technological competition. However, to effectively identify bottleneck technologies using the industry chain approach, one must first identify all relevant enterprises within the industry, develop the various stages of the industry chain, and then utilize patent information to recognize monopolistic technologies within the industry.

What are the technological development characteristics of these bottleneck technologies? Technological development characteristics primarily refer to the changes that occur in the process of technology transfer among users. This discussion aims to explore the historical development and direction of these technologies, primarily by examining mutual patent citations to trace the main paths of technological development. Subsequently, it involves tracing the technological development trajectory over time to reveal patterns and trends in technology development.

This paper is systematically organized into many sections. Section 2 presents a comprehensive review of existing literature on bottleneck technologies in the electronic manufacturing industry, emphasizing the role of patent information in identifying bottleneck technologies and understanding their diffusion and evolution. Section 3 delineates the data resources and research methodologies employed in this study. Section 4 is dedicated to the identification of bottleneck technologies in the electronic manufacturing sector. Section 5 explores the diffusion, evolutionary characteristics, and trends of these identified bottleneck technologies. The final section culminates in a discussion, conclusion, and proposes directions for future research in this domain.

## 2. Literature review

### 2.1 Key technologies, bottleneck technologies and their difference

Before bottleneck technology, the term key technology was more commonly used. Previous research in the electronic manufacturing industry has encompassed various aspects. For instance, in their study of Taiwan’s semiconductor foundry industry, Chen et al. (2009) emphasized the increasing significance of technology in achieving business success [8]. They proposed that, for the industry’s sustainable development, a well-considered plan for selecting and developing key technologies should be established. Additionally, Graf (2012) delved into emerging key technologies in information technology and semiconductors, studying inventor networks in these domains [9]. Furthermore, Chang (2019) utilized patent analysis to unveil trends and key technologies in 5G photonics, revealing close associations with optical components and semiconductor devices [10]. Their research culminated in the development of a patent technology network model, facilitating insights into the development trends of 5G photonics technology and offering valuable references for government efforts to promote these emerging technologies.

The concept of bottleneck technology started to attract attention, particularly following the 2018 "ZTE incident". Firstly, it is crucial to differentiate bottleneck technology from key technology. Key technology is defined as technology that fulfills an essential and irreplaceable function within a system, industrial chain, or technological domain. In contrast, bottleneck technologies typically exhibit a more monopolistic nature. This technology is typically under the control of a nation or a single enterprise, which gives them a significant advantage over competing products and allows them to dominate the market. The technology supplier exerts impact in this particular scenario. Consequently, bottleneck technology is always considered a key technology. Only when a specific key technology is monopolized by a very small number of companies does that key technology become a bottleneck technology. Furthermore, it is essential to acknowledge the distinction between bottleneck technology and disruptive technology. Disruptive technology pioneers innovative pathways and challenges conventional or established technologies, while bottleneck technology boasts a long-standing record of development.

### 2.2 Patent as indicators for identifying technologies in industries

Patent analysis is a highly effective method for technology discovery, encompassing the selection, identification, organization, and subsequent utilization of various information technology tools, including bibliometrics and content analysis. The process involves the statistical comparison, extraction, and analysis of the information elements found in patent documents. This process facilitates the exploration of intelligence and comprehension of the dynamic evolution of technology. At present, patent analysis is considered one of the most effective methods for accomplishing these objectives. Tian et al. (2022)utilized an enhanced Latent Dirichlet Allocation (LDA) model to extract technical themes from standard essential patents. They identified key technologies by evaluating the weight of each topic [11]. Wang et al. (2022)developed a "technology-efficiency" matrix by employing Subject-Action-Object (SAO) semantic analysis of patent data to identify the unexplored technology domains related to graphene [12]. Gao et al. (2021) employed the count of primary International Patent Classifications (IPCs) and the proximity among IPC technologies in patents to elucidate the evolutionary patterns of 5G network technology integrating blockchain [13]. In the analysis, the importance of technologies was evaluated based on the following node topology characteristics: (1) degree, (2) hits, (3) pagerank, (4) node betweenness, (5) eccentric distance, and (6) clustering coefficient. Their study encompassed the utilization of applications, architectural diagrams, and methods for patent analysis. The analysis included an examination of the prominent countries and companies involved in the advancement of 5G network technology. It aimed to pinpoint obstacles in technological progress and emphasize the comparative advantages and disadvantages. Zhang et al. (2018) conducted statistical analyses utilizing Beijing IncoShare patent database. They examined the correlation between IPC codes and specific technologies and products within the industry to investigate the progression, geographical dispersion, variations in types, and IPC of patent applications in China’s offshore wind power sector [14]. Shin et al. (2013) utilized patent citation network analysis to identify key technologies, forecast the future growth paths of these technologies, and assess their risk-adjusted future performance. Centrality and brokerage of citation networks were utilized to assess the direct and indirect flow of technological knowledge, aiming to pinpoint the pivotal Image and Display technologies [15].

### 2.3 Characteristics of technological development

The characteristics of technological development primarily pertain to the alterations that take place in a technology throughout its developmental process. In addition to investigating technology transfer across various countries or regions, the evolution and collaboration of technology stand out as crucial research areas. Patents serve as a crucial repository of technological knowledge, encompassing a significant volume of interconnected information, including citation relationships and identical technical codes. Therefore, patents can offer a comprehensive insight into the process of technological diffusion and the evolutionary dynamics associated with patents. The predominant approach in general research involves utilizing citation relationships. Zhou et al. (2016) conducted a comparative study of the knowledge bases of Asian and European wind turbine enterprises, focusing on patent trajectories, networks, and globalization of knowledge using patent analysis [16]. Their research centered on three aspects: the trajectories of key technologies, external knowledge networks, and the globalization of knowledge application. The study revealed that European enterprises continue to lead the industry, and leading Asian enterprises are unlikely to create new paths to disrupt existing enterprises in the near future. Wang et al. (2018) utilized the PatentLens database to develop patent citation networks for Chinese solar photovoltaic and solar water heater technologies [17]. They also examined the trajectories of key technologies and collaboration types. The study found that relying on China’s traditional industrial model can establish a short-term market development model but may not foster a long-term internationally leading innovation model. International leadership in innovation can only be achieved through the comprehensive application of integrated science, technology, and innovation models. Furthermore, Jiang et al. (2022) utilized patent citation analysis based on patent data published from 1970 to 2019 to explore international patterns of artificial intelligence technology diffusion [18]. They established a connection between technological innovation capability and the degree of international technology diffusion. They categorized different countries/regions into three groups: leading, middle, and backward countries, based on their technological positions in this context.

## 3. Materials and methods

### 3.1 Data source

The enterprise data utilized in this study was sourced from BvD’s Orbis corporate database, which encompasses comprehensive business information for major enterprises worldwide. Patent data was obtained from Derwent Innovation (DI) database by Clarivate Analytics. The utilization of shareholding relationship data from Orbis database allowed for the extraction of holding enterprise names. These names were subsequently optimized for patent retrieval purposes, ensuring the comprehensiveness of the analysis. The primary objective of this research was to identify oligopoly technologies within the electronic manufacturing industry. As such, the related patents must have been developed and protected on a global scale. Consequently, the selection of patents for analysis was limited to U.S. patents.

In the Orbis database, it is recommended to initiate the search by looking for core industry enterprises. According to NACE Rev. 2 industry standards, the search for enterprises should be classified under industry codes 26 (i.e., manufacturing of computer, electronic, and optical products) and 27 (i.e., manufacturing of electrical equipment). Subsequently, it is advisable to explore enterprises within the industry that are either upstream or downstream. In general, every industry encompasses production equipment, materials, and testing. Therefore, in enterprises categorized under industry codes 20 (i.e., manufacturing of chemicals and chemical products) and 28 (i.e., manufacturing of machinery and equipment nec), the companies should be identified using search terms such as "semiconductor", "electronic devices", and "chips" within the fields of "industry group description", "main business description", and "products and services". A total of 1,395 enterprises were identified, restricted to those with annual revenues surpassing 1,000 million USD. These enterprises encompass prominent industry participants such as Hitachi, Intel, Applied Materials, and Carl Zeiss, among others. It is crucial to acknowledge that numerous enterprises have a diverse range of products and operate across multiple industry sectors, as previously indicated.

Utilizing equity relationship data from the Orbis database, this study extracted and employed enterprise names as controlled variables for optimized enterprise names in patent retrieval. The time frame chosen for patent retrieval ranged from 2018 to 2022 (application year), with the retrieval process taking place in March 2024. Among the 1,395 enterprises, there is a collective total of 370,065 patents. A cumulative of 225 firms possessed over 100 patents. See the first column of S1 Table in the appendices.

### 3.2 Research methods

**Fig 1:**
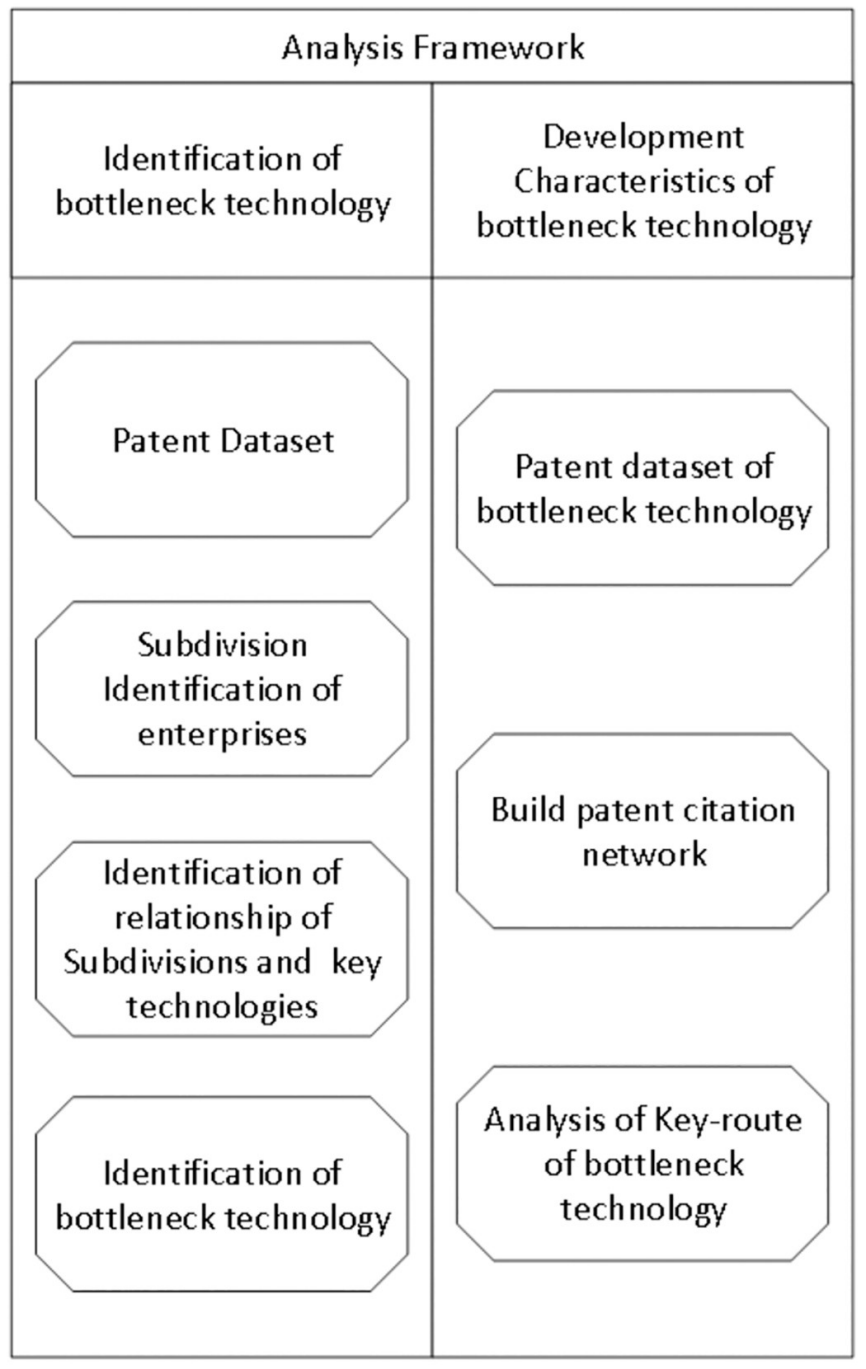
Proposed analysis framework.

The research analysis is primarily divided into two integral parts, as illustrated in Fig 1. The initial segment of the study entails the identification of bottleneck technologies within the electronic manufacturing industry. To initiate this process, we first identify the main technical areas of these enterprises by analyzing the technology codes in their patent datasets in order to acquire the industry chain information where these enterprises are located. Code co-occurrence analysis is then employed to conduct community network analysis, facilitating the extraction of interrelationships among various segments of the industry chain. Ultimately, bottleneck technologies and their affiliated enterprises are identified based on patent strengths. The second phase of the analysis is dedicated to comprehending the diffusion, evolutionary characteristics, and trends of these bottleneck technologies. This involves developing a citation network using citation information from patents. Through the implementation of key path analysis methods, the study identifies critical paths in the technological development and evolution, offering insights into the origins, diffusion dynamics, and development trends of these technologies. Subsequent sections provide a more detailed elucidation of the analysis process.

#### 3.2.1 Identification of technology field topics and enterprise competitive domains

In commercial databases, although it is possible to identify the involved enterprises, often, information regarding their areas of technological expertise and their specific sub-industries is not readily available. Therefore, it becomes necessary to identify these details using patent data. This is because enterprises today are involved in numerous technology fields, often having various subsidiaries. Consequently, it is essential to categorize these enterprises into the various segments of the industry chain under investigation. In this research, a social network is developed for identification using the unique DI database’s Derwent Manual Codes (referred to as technology codes hereafter). These technology codes are manually assigned, focusing on practical applications, and are more precise compared to International Patent Classification (IPC) and Cooperative Patent Classification (CPC). The specific implementation involves initially creating a collection of enterprise patent technology codes, as illustrated in Eq (1), and then calculating Pearson correlation coefficient to determine their similarity. Pearson correlation coefficient is a significant metric used to assess the resemblance between datasets. This coefficient is commonly utilized in assessing the similarity of patent data. Deng et al. (2023) assessed the similarity of expanded patent phrases by employing Pearson correlation coefficient [19]. Zhu et al. (2024) employed Pearson correlation coefficient to assess the resemblance among patent phrases [20].


$$\text{A}=\left[\begin{array}{c} Tech.\;Code1\\ a_{1}\\ \vdots \\ a_{i} \end{array}\begin{array}{c} Tech.\;Code2\\ b_{1}\\ \vdots \\ b_{i} \end{array}\begin{array}{c} \ldots \\ \ldots \\ \ldots \\ \ldots \end{array}\begin{array}{c} Tech.\;CodeN\\ x_{1}\\ \vdots \\ x_{i} \end{array}\right]$$ (1)

$$r=\frac{\sum_{i=1}^{n}\left(X_{i}-\bar{X}\right)\left(Y_{i}-\bar{Y}\right)}{\sqrt{\sum_{i=1}^{n}{\left(X_{i}-\bar{X}\right)}^{2}}\sqrt{{\sum_{i=1}^{n}\left(Y_{i}-\bar{Y}\right)}^{2}}}$$ (2)

In Eq (1), A represents the collection of all enterprise technology codes, where a_i_, b_i_, …, x_i_ represent the number of patents that enterprise i holds within a particular technology code. The primary purpose of calculating the correlation coefficient among enterprises within the industry is to determine which specific subdomains these selected enterprises belong to within the industry. This information is crucial for identifying the upstream and downstream relationships within the industry and pinpointing the key technology fields in the target industry. It provides guidance for the subsequent identification of industry key codes.

#### 3.2.2 Identification of industry key technologies

The identification of pivotal technologies within the industry serves as the basis for identifying bottleneck technologies. This study predominantly employs the community network of technology codes to pinpoint essential technologies within the industry. Building a network of patents is a prevalent approach for identifying crucial technologies. Construction processes typically rely heavily on keywords and IPC codes to identify key technologies. Subsequently, key technologies are determined by calculating specific characteristic indicators within various clusters. Wang et al. (2023) employed the patent clustering method to develop Patent-IPC matrix and ascertain the pivotal core technologies utilizing IPC [21]. Woo et al. (2016) identified the fundamental core technology in robot technologies by creating a co-occurrence matrix of keywords from various patent specifications. In contrast to previous research, this study employed DWPI technology code, which is manually indexed and aligns more closely with general technical descriptions than IPC, offering higher reference value [22].

**Fig 2:**
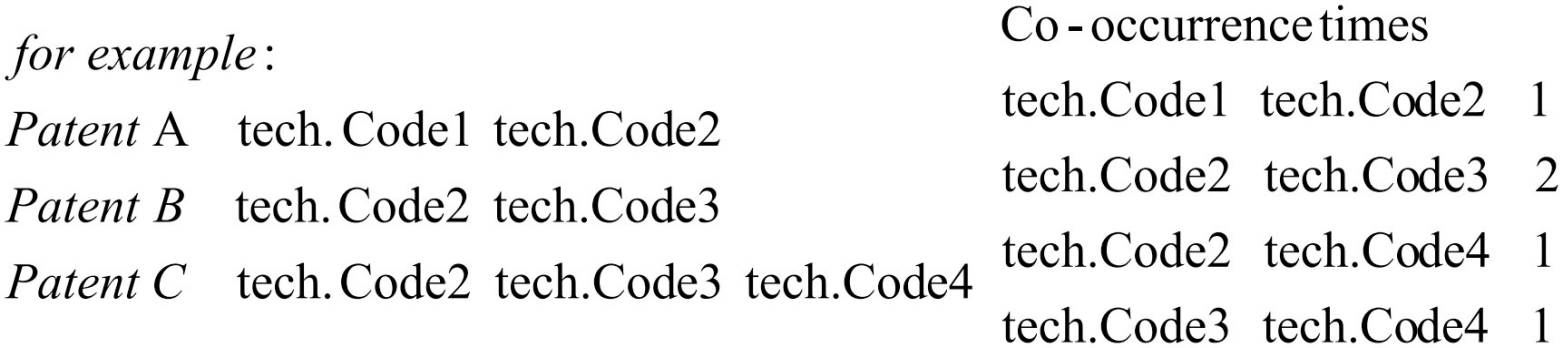
Illustration of patent technical code co-occurrence frequencies.

First, develop a co-occurrence network of patent codes by calculating the co-occurrence frequency of any two patent technology codes within all samples, as shown in Fig 2;

Second, use clustering algorithms with developing technology code clusters. Various methods are available for clustering. This study employs the Fast Unfolding method [23]. This method partitions communities based on the weight values between any two points, optimizing the community modularity (Q). Modularity, in simple terms, involves grouping densely connected points into one community, resulting in an increased modularity value. The final partition with the highest modularity is considered the optimal community division. Several software tools, such as Pajek and Gephi, can perform this calculation. Typically, a Q value within the range of 0.3 to 0.7 indicates a significant and structurally advantageous community division [23]. In this research, the weight values calculated in the first step for the co-occurrence of any two patent technology codes are used to compute the Q value in the software.

Third, to identify key technologies two characteristic indicators are used. At first, identify central codes within the patent communities based on Degree Centrality, as demonstrated in Eq (3). The code with the highest centrality is the code with the highest degree of correlation in the cluster, generally representing key technologies within a technical region.


$$DC_{i}=\frac{k_{i}}{N-1}$$ (3)

In Eq (3), *DC*_*i*_ indicates the degree centrality; *k*_*i*_ indicates the number of edges that node *i* is directly connected to other nodes; *N* is the number of nodes; and *N*−1 indicates the number of edges where node *i* is connected to other nodes.

**Fig 3:**
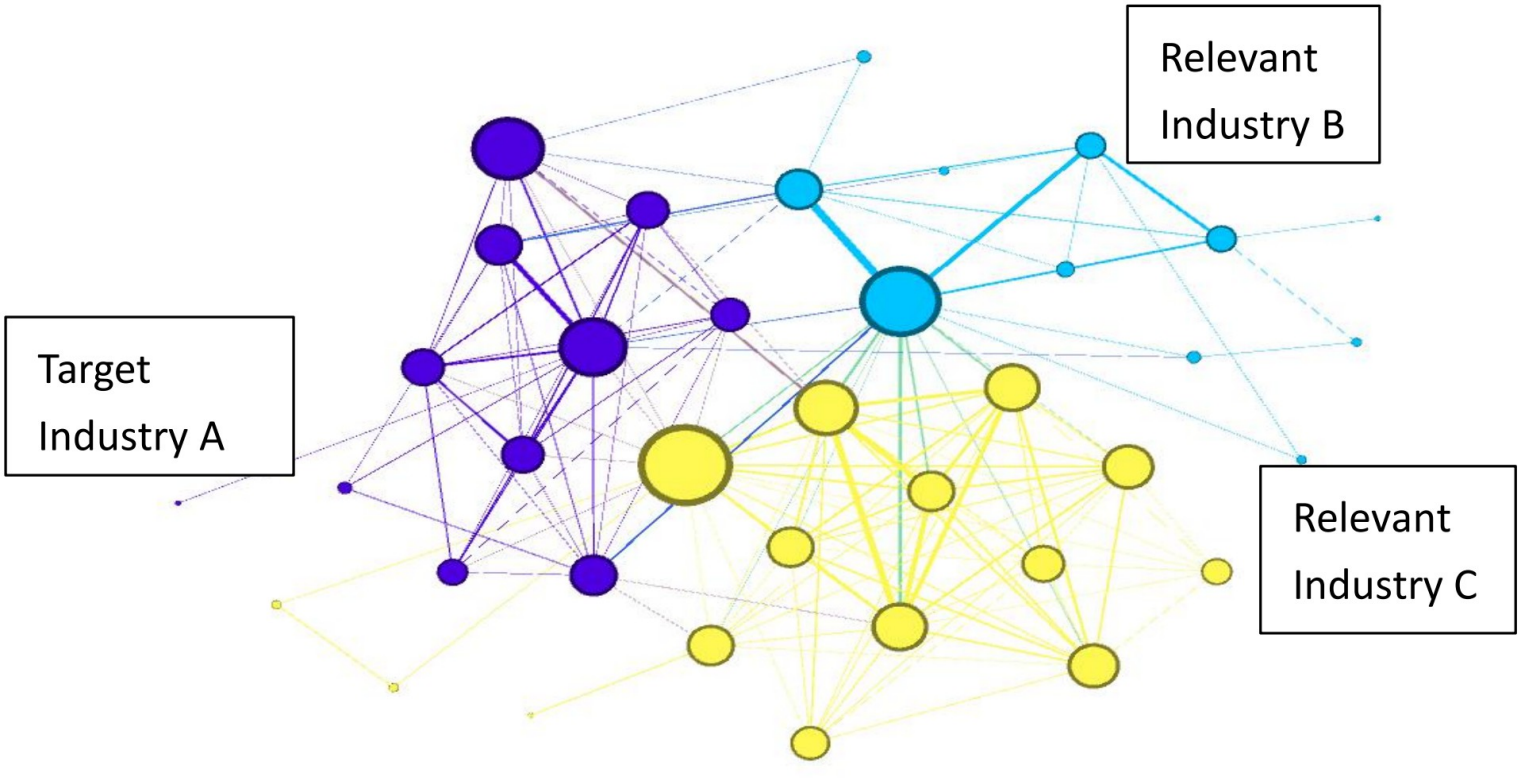
Schematic diagram for identifying key industry technology codes.

In addition, we also need to identify nodes between different technical areas, which are also key technologies because they can link different technical areas and are very important for the industrial chain. For example, as illustrated in Fig 3, the patents in the concentrated area of technology codes for Target Industry A are represented in purple, whereas the blue and yellow areas represent patent codes related to Industries B and C, which are associated with Target Industry A. To recognize the technologies involved in Target Industry A within Industries B and C, it is necessary to identify the associated points, thereby identifying critical technologies and components within Industries B and C that may pertain to Target Industry A.

This connecting point can be understood as nodes with a higher Betweenness Centrality in two technical communities. In other words, if there are more shortest paths passing through a node between two communities, it indicates that this node has a more pronounced intermediary effect between the communities. Therefore, by calculating the number of shortest paths passing through a node, the study can identify the connecting code points between different technical communities. Betweenness Centrality is defined as shown in Eq (4).


$$BC_{i}=\frac{1}{(N-1)(N-2)/2}\sum_{s\neq i\neq t}\frac{n_{st}^{i}}{g_{st}}$$ (4)

In Eq (4), *BC*_*i*_ indicates the betweenness centrality; *n*^*i*^_*st*_ indicates the number of shortest paths from *s* to *t* through node *i*; *g*_*st*_ indicates the number of shortest paths connecting *s* and *t*; and *N* is the number of nodes.

#### 3.2.3 Identification of bottleneck technologies

Bottleneck technologies tend to exhibit a higher degree of monopolistic characteristics in comparison to key technologies. This technology is typically dominated by a single entity or a small number of enterprises, possessing a significant competitive edge over other similar businesses, thereby monopolizing the entire market. Consequently, the ultimate stage of the analysis involves determining the key technologies that hold monopolies. Ernst utilizes the technology share indicator to signify an enterprise’s advantageous position in patents, a method that has gained extensive utilization in various contexts [24]. The technology share indicator is primarily denoted by Technological Specialization Advantage (TSA) [25].


$$\text{TSA}:\;TSA=PA_{iF}/\sum_{i=1}^{n}PA_{F}$$ (5)

In Eq (5), *PA*_*iF*_ represents the number of patent applications by enterprise *i* in the technology code field *F*. Based on the identified industry technology codes, this study uses this indicator to calculate the technology-advantage, in order to determine and recognize an enterprise’s dominant technologies and find its monopoly position in the industry’s technology landscape.

#### 3.2.4 Patent citation network and key path analysis of bottleneck technologies

The citation relationships between patents serve to highlight the scientific underpinnings of technology and reveal the inheritance and accumulation relationships between technologies [26–29]. Thus, a patent citation network based on these citation relationships can provide development characteristics of a technology and also insights into the technological context of a specific field [30]. In this study, the development of a citation network was used to identify key paths in technology diffusion [31]. This approach not only allows for tracking and describing the flow of technology from a patent perspective but also offers a tool and method for understanding diffusion paths within specific technology fields. The concept of key-route main path analysis originates from main path analysis. The main paths represent critical routes within a citation network used to identify key knowledge flow paths. Hummon et al. [30] argued that the main paths encompass the most important trajectories within a citation network and employed a ’priority search’ algorithm to trace the most crucial paths. Building upon this, Batagelj et al. [32] proposed enhancements to main path analysis, including Search Path Count (SPC), Search Path Link Count (SPLC), Search Path Node Pair (SPNP), and Node Pair Projection Count (NPPC). They considered SPC method superior to others, and many researchers have applied this method to study the developmental trajectories within various technological fields. Liu et al. (2011) further extended main path analysis to propose the key-route main path analysis method [33]. Key-route main path analysis simplifies complex networks into straightforward paths by eliminating less important information, retaining only the most critical paths. This method simplifies the discovery of research and development trajectories in technology and offers a more straightforward way to compare different technological paths. The steps involved are as follows: ① Identifying the links with the highest SPC values (traversal counts) within the network. ② Searching from the endpoint of the target link to find the most efficient forward path, and then searching from the starting node of the same link to find the most effective backward path. ③ Combining the results from forward and backward searches into a single path. ④ Merging the paths from both ends of the link to form a key path. In this study, Pajek was used to calculate SPC values and derive the key paths.

**Fig 4:**
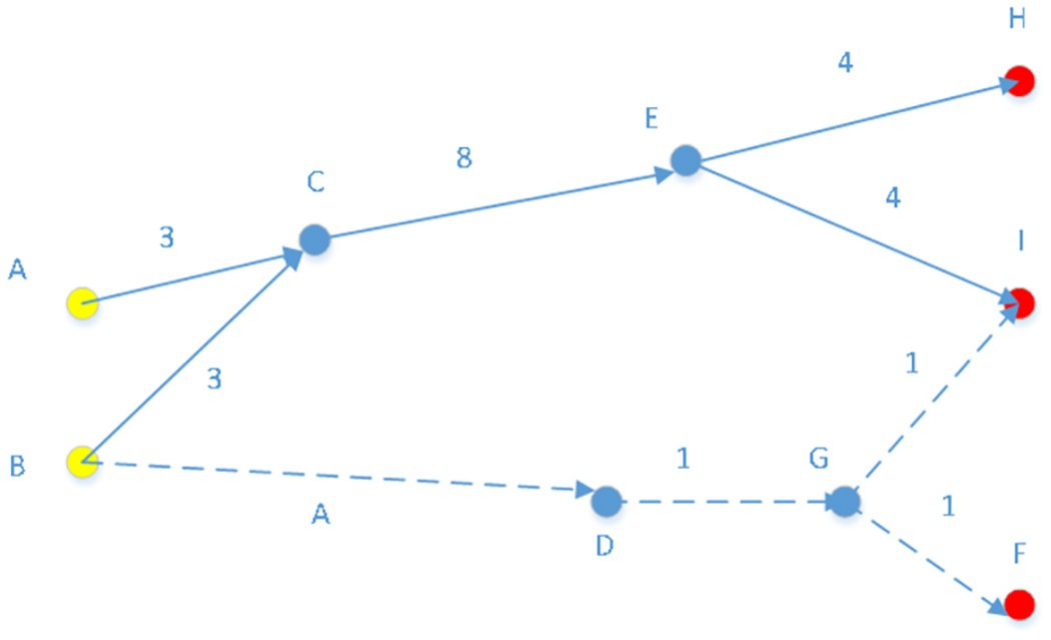
Depiction of key path in knowledge diffusion.

To facilitate comprehension, consider Fig 4 as an illustrative example. In this figure, nodes A and B represent origins, while F, H, and I are terminal nodes. Among all citation links, the pair <C, E> exhibits the highest SPC (Shortest Path Count) value, indicating its pivotal role in the process of knowledge diffusion. Consequently, the link <C, E> is identified as the critical path. By analyzing the connections extending forwards and backwards from this link, the study can delineate the primary route of knowledge transfer, as depicted by the solid lines in Fig 4.

Subsequently, the study defines two metrics to analyze the evolution of technology. The first metric is the number of key path nodes in the main path, where each node represents a significant patent in the technological development process. A higher count of patents indicates that the development of this bottleneck technology requires a longer period and more initial accumulation. The second metric is the number of patent classification code categories contained in each node (i.e., each patent), which corresponds to the number of IPC code categories. A higher number of code types suggests a broader functional scope for this technology, requiring knowledge from various fields and a higher level of technological complexity. Furthermore, this paper also analyzes changes in IPC codes throughout the entire technological development process and discuss the trends in technological development.

## 4. Results

### 4.1 Identification of bottleneck technologies

#### 4.1.1 Subdivision of technology field topics and enterprise competitive domains

After selecting the relevant enterprises, the study initially categorizes the enterprises based on their technology fields, following the procedure described in Section 3.2.1 of this paper. The resulting correlation matrix is shown in Table 1. The complete version has been included in the appendices. (see S1 Table). According to the range of correlation coefficients, the major patent holders can be divided into 4 domains (chips, equipment, materials and application), with a total of 12 groups, as shown in Table 2. Depending on the industry field, the range of correlation coefficients varies. The chip domain has the highest correlation, especially in the first group, where several enterprises have highly concentrated patent portfolios. In the third group, the correlation coefficients have a broader range, mainly due to the cross-similarity within the enterprises in the group, forming several smaller similar clusters. However, the similarity to enterprises outside of these small clusters is lower, indicating that while these enterprises are all in the chip industry, there are significant differences in the types and applications of chips they focus on. Equipment manufacturers have lower correlation compared to chip enterprises but are divided into 3 subcategories, forming a hierarchical structure. Materials-related enterprises have the lowest correlation. The main reason for these differences in similarity is the different domains in the electronic manufacturing industry that each enterprise operates in. The chip industry has higher overall uniformity in general technology but differences in data processing functions, leading to higher similarity with slight variations. Equipment enterprises often engage in the research and production of equipment across multiple domains, leading to lower similarity compared to chip enterprises. Material enterprises deal with a broader range of domains than equipment enterprises, resulting in even lower similarity. Through this analysis, it is clear that the main technologies in the electronic manufacturing industry come from chips, equipment, and materials. However, these results do not yet identify the key technologies within the industry, as further associations within the subdivided industry technology fields are needed to discern the industry’s advantageous technologies.

**Table 1:** Correlation coefficient matrix of enterprise patent code (partial display due to space constraints).

Name of enterprise	HUAW-C	APPY-C	ASUS-C	HOYA-C	ITLC-C	MCRN-C	QUAT-C
HUAW-C	1	—	—	—	—	—	—
APPY-C	.858	1	—	—	—	—	—
ASUS-C	.703	.692	1	—	—	—	—
HOYA-C	.044	.073	.000	1	—	—	—
ITLC-C	.696	.757	.345	.066	1	—	—
MCRN-C	.251	.027	.054	.012	.520	1	—
QUAT-C	.115	.216	.202	.037	.294	.137	1

**Table 2:** Subclassification of major patent holders in different technology fields.

No.	Field	Patent holders	Correlation coefficient range
1	Chip-1	ITLL-C, FUIT-C, NIDE-C	0.8 ~ 0.9
2	Chip-2	HITA-C, ADMI-C, MOTI-C, INFN-C, NVID-C,	0.5 ~ 0.6
3	Chip-3	TRDN-C, SNKE-C, XILI-C, TEXI-C, SONY-C	0.2 ~ 0.8
4	Chip-4	MCRN-C, HYNX-C, WDIG-C, WINB-C, TOOR-C	0.6 ~ 0.8
5	Equipment-1	ZEIS-C, ASML-C, KLAC-C	0.3 ~ 0.5
6	Equipment-2	SHIA-C, AMKR-C, ASEG-C	0.7 ~ 0.9
7	Equipment-3	TKEL-C, ADEN-C, ROCW-C	0.4 ~ 0.5
8	Material-1	TORA-C, ASAE-C, NITL-C, WACK-C, TOKQ-C,	0.3 ~ 0.6
9	Material-2	TKEL-C, APMA-C, LRES-C	0.3 ~ 0.8
10	Application-1	INLX-C, BOEG-C	0.8 ~ 0.9
11	Application-2	ACER-C, ASUS-C, PEGA-C	0.5 ~ 0.7
12	Application-2	APPY-C, BBKO-C, GDOP-C, HUAW-C	0.8 ~ 0.9

#### 4.1.2 Identification of key industrial technologies

Using Gephi, the co-occurrence counts of any two patent codes from the entire sample were brought in to calculate the Q value, resulting in Q = 0.567. This value falls within the range of 0.3 to 0.7, signifying significant community division. The clustering results are illustrated in Table 3 (Column 1), where 11 clusters are identified. Additionally, for each area, central technology codes within the community were determined based on degree centrality calculations. These codes were then categorized into specific technology fields according to the explanation provided in the technology code manual, as presented in Table 3, Column 3.

**Table 3:** Core technology codes in technology communities (listing the most important technology codes (Top 5)).

Cluster No.	Technology field	Core codes	Intermediary technology code
1	Process equipment	L04-F03, U11-C09X, L04-C12, U11-C05C, U11-C05B4	-
2	Polymers, catalyst equipment, processes	A12-W11A, F02-C01, N06-F, J04-E04A, N07-L02C	F02-C01, N06-F, J04-E04A, A04-E08, A11-C05A
3	Material testing, circuit testing	U11-F01C3, U11-F01B3, S01-G02B, S03-E06B1, S01-G02B1	U11-F01C3, U11-F01B3, S02-A03, S01-G02B, U11-F01B1
4	Data processing, information transmission of electronic devices	T01-S03, T01-N02A3C, W01-C01D3C, W01-C01P2, T01-C03C	A12-E07C, L04-F03, L04-C11C, L04-E15, U14-A03B7
5	Storage devices	U14-A03B7, T01-L01, T01-C01, U14-A03B4, T01-G03	T03-A08A1C, T01-K, T01-H01B4, U22-A02D, U22-A04C
6	Automatic control system	P62-E, P62-F, T06-D07B, T01-J07B, X25-A03E	Q42-A10, T01-J07B, Q64-C01, X22-J05, T01-J07D1A
7	Voltage control system	X12-J01A, U24-D01A, X12-J02A, U24-D02A, V02-G02A2	M27-A04, U21-B01B, X13-H01D1C, L03-B02J, V06-N03
8	Printing system, imaging system	P84-A02, G02-A04A, S06-K99C, V06-V01E, S06-K03	T01-S03, W01-C01D3C, W01-A06C4, T01-C03C, T01-J10D
9	Electric vehicles, battery systems	X16-B01F1, L03-E03, L03-H05, L03-E01B5B, X21-A01F	T01-S03, W01-C01D3C, W01-A06C4, T01-C03C, T01-J10D
10	Pharmaceutical processes, diagnostic equipment, microbiology, natural material testing equipment	D05-H99, D05-H09, C14-S18, D05-H16B, C14-U01	T01-S03, W01-C01D3C, W01-A06C4, T01-J05B4P, T01-C03C
11	LED Technologies	U12-A01A7, U12-A01A1E, U14-K01A1B, L04-E03, W03-A08J	P73-V16, A12-S05L, E06-H, E05-E01B, G03-B04

The electronic manufacturing industry’s technology is mainly divided into two parts: the process equipment area centered in Cluster-1 (semiconductor devices manufacturing process equipment) and the semiconductor component products centered in Cluster-4 and Cluster-5 (major semiconductor products—processing chips and storage chips used in the industry). Cluster-1, Cluster-4 and Cluster-5 are the largest in terms of cluster and the most densely populated with technology codes. They constitute the core areas of electronic manufacturing industry technology. Moreover, these two parts have numerous co-occurring and associated technologies, as well as significant co-occurrence with other areas. In other words, the technologies within these two parts have the greatest influence on other clusters and form the foundation of the industry.

Prior to developing the key intermediate technology codes across the entire industry chain, it is necessary to differentiate the upstream and downstream sectors within the subdivided technology fields. Cluster-2 and Cluster-3 primarily involve the basic materials and testing equipment for semiconductor production, and they mostly co-occur with Cluster-1, which uses their technologies. Therefore, the intermediate technology codes are present in the upstream Cluster-2 and Cluster-3. On the other hand, Cluster-4 and Cluster-5 are the primary clusters for semiconductor component products, and its upstream technologies are mainly found in Cluster-1, which pertains to semiconductor component manufacturing processes and equipment. The key constraining technologies for Cluster-4 and Cluster-5 are also located in Cluster-1. In contrast, in the other areas, namely Cluster-6 to Cluster-11, the focus is on the different appliction of the electronic manufacturing industry. Their associated relationships primarily point to Cluster-4 and Cluster-5, indicating that they predominantly utilize electronic manufacturing industry products (such as chips and memory) as their components. Cluster-4 and Cluster 5, representing the semiconductor component products, play a crucial supporting role for the other six clusters. Therefore, its core intermediate technology codes are located in Cluster-4 and Cluster-5.

Following the method described in Section 3.2.3 of this study, starting from each respective core code area, the betweenness centrality of nodes in other communities was calculated to identify the core intermediate technologies in their associated areas. This process was used to determine the core technologies upstream in the industry chain, as well as to find the core technologies constraining downstream areas. The corresponding codes for these core technologies were included in Column 4 of Table 3 to create a comprehensive panorama of the industry’s patent code relationships.

#### 4.1.3 Analysis of core technology codes in the electronic manufacturing industry

From Table 3, it can be observed that the main technologies in the electronic manufacturing industry are concentrated in process equipment (Cluster-1) and electronic devices including devices for data processing and information transmission (Cluster-4) and devices for storage (Cluster 5). From an industry perspective, the core technologies in process equipment include U11-C05 (Semiconductor Etching Technology), U11-C04 (Lithography Technology), L04-F03 (Semiconductor Component Assembly Technology), and L04-C12 (Semiconductor Insulation Passivation Technology). These primarily involve upstream semiconductor component production processes and equipment technologies used in the electronic manufacturing industry. In the midstream and downstream of the industry, the main core technologies include T01-S03 (Software Technology for Digital Computers), T01-N02 (Communication and Control Technology for Digital Computers), T03-C03 (Data Interchange Technology), W01-C01 (Communication and Data Transmission Technology). This indicates that these core technologies are related to chip technologies used for data processing and storage.

Additionally, auxiliary technologies in the electronic manufacturing industry encompass chemical and chemical engineering technologies used in the production process (Cluster-2 and Cluster-3), primarily including A12-W11 (Reverse Osmosis Membrane Technology for Purification), F02-C01 (Semiconductor Cyclization Process Technology), J04-E04 (Catalyst Technology), N07-L02 (Catalyst Application Technology), as well as production testing technologies, including U11-F01 (Physical Property Measurement Technology), and S01-G02 (Failure Detection Technology), S03-E06 (Material Property Testing Technology). Moreover, the core patent codes in these two auxiliary technology clusters and the intermediary patent codes that are most prominently connected to the main area exhibit a high level of consistency. Except for A04-E08 (Polytetrafluoroethylene Technology) and S02-A03 (Optical Measurement Technology), which appear independently as intermediary technologies, the core and intermediary technology codes in other categories are essentially the same. This indicates that these auxiliary technologies are highly aligned with semiconductor component production processes and are crucial for production.

Conversely, in Cluster-5 to Cluster-11, the core technologies and intermediary technologies are fundamentally different. For instance, technologies like X21-A01 (Electric Propulsion Technology) and D05-H09 (Microbiological Detection Technology) are prevalent in these areas. This indicates that the core technologies in these areas are primarily related to the practical application of semiconductor products as components, rather than being directly associated with semiconductor technology itself. Consequently, the key intermediary technologies involving semiconductors are quite similar across these areas and mainly include T01-S03 (Software Technology for Digital Computers), W01-C01 (Wireless Data Transmission Technology), T01-C03 (Digital Interaction Technology), T01-J07 (Data Processing Technology), and T01-M06 (Data Processing Architecture Technology).

#### 4.1.4 Identification and analysis of bottleneck technologies

To distinguish and select enterprises and technologies with technical monopolies, this study uses indicator TSA provided in Section 3.2.4 of this paper, combined with the technology fields identified in the previous section. For ease of reading, areas with values greater than 0.3 are highlighted in red, as shown in Table 4.

**Table 4:** Representative sample of technology codes across various enterprises.

Technology code\Enterprises	MCRN-C	LRES-C	NITL-C
U11-C18C	0.020	0.004	0.129
A12-E01	0.151	0.017	0.065
L03-H03A	0.037	0.003	0.100
L03-J02	0.418	0.003	0.000
U14-A03B4	0.026	0.057	0.104
L03-G05	0.048	0.001	0.008
U11-D01A	0.037	0.004	0.016
U11-D03B3	0.049	0.044	0.086
L03-H03	0.066	0.003	0.006
S01-G02B	0.046	0.039	0.004
T01-J07B2	0.317	0.007	0.000
U11-C18B5	0.354	0.001	0.000
U14-A03B7	0.056	0.000	0.000
U12-D02A	0.094	0.036	0.004
L04-C13B	0.028	0.016	0.008
U11-C01J2	0.307	0.017	0.000
L04-E15	0.027	0.009	0.004
U11-F01C3	0.145	0.000	0.002
U12-Q	0.046	0.010	0.006

**Fig 5:**
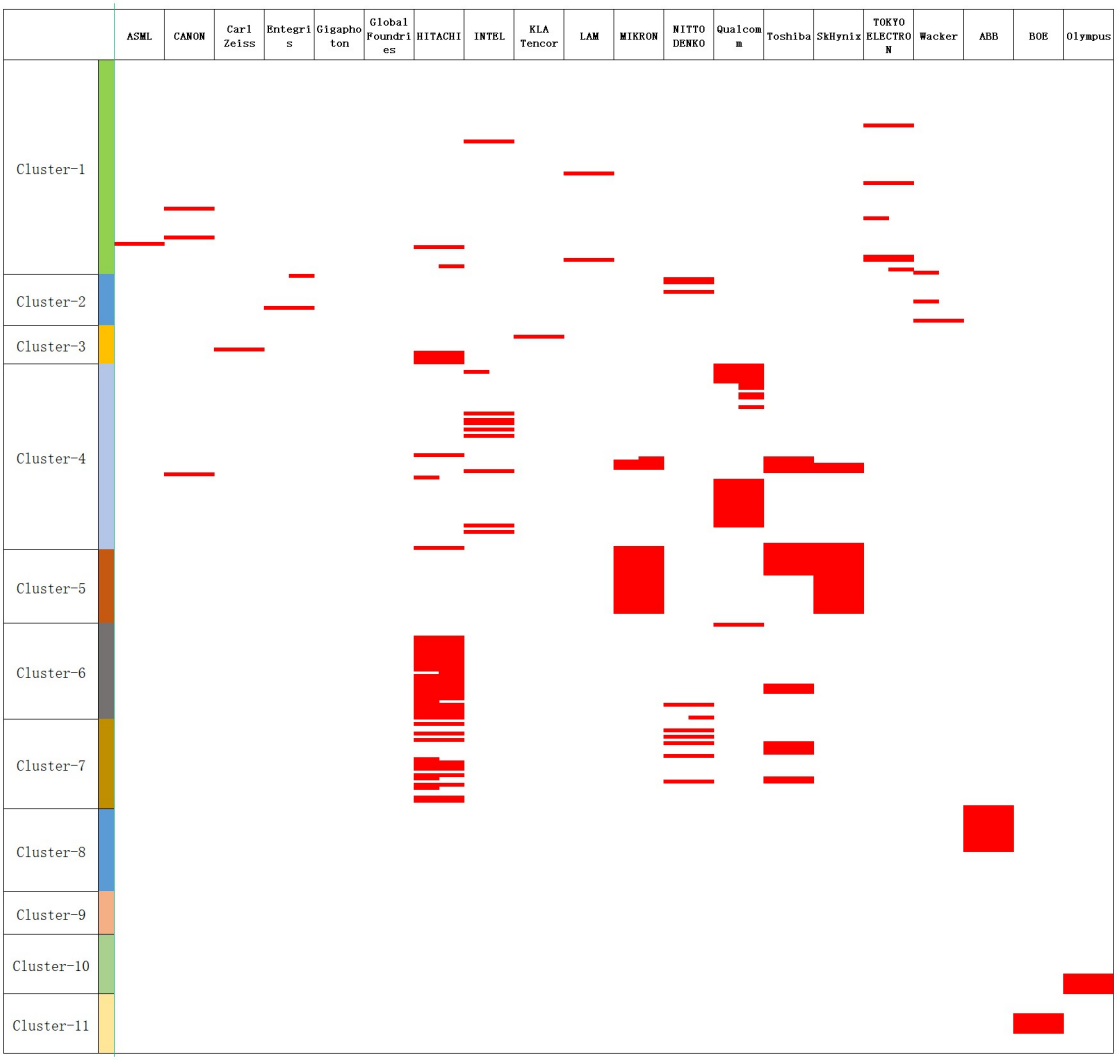
Technology—Enterprise advantageous map (partial display due to space constraints).

Based on the calculated competitive advantages of enterprises in different fields, a two-dimensional chart of enterprise-technology fields (composed of technology codes from different fields) can be established to identify advantageous enterprises in each technology field, including core technology fields and key intermediate technology fields. Due to the large number of enterprises, the Excel file has been reduced in size for ease of reading, as shown in Fig 5.

In Fig 5, the characteristics of advantageous technologies vary across different subfields. In the clusters of process equipment and material (Cluster-1, Cluster-2, and Cluster-3), there are a relatively larger number of enterprises with advantageous technologies, and the technologies are more dispersed. Different enterprises possess proprietary technologies in different domains within this field. In the main semiconductor component product clusters (Cluster-4 and Cluster 5), there are instances of technology concentration among enterprises. Many enterprises have a significant share of patents, resulting in broad coverage. In the clusters of applications (Cluster-5 to Cluster-11), a similar phenomenon is observed, with a concentration of patents among a few enterprises. However, due to many large enterprises in the industry both manufacturing semiconductor components and downstream products, and enterprises exclusively manufacturing downstream products not being included in this analysis, it creates an appearance of dominance by a single enterprise. Moreover, the internal codes within this field are not within the scope of this study. As indicated by the previous analysis, the critical technologies in this segment depend on those in Cluster-4 and Cluster-5. Therefore, the subsequent analysis focuses specifically on Cluster-1 to Cluster-5.

Table 5 summarizes the key technologies in the electronic manufacturing industry and the core intermediary technologies that constrain the industry upstream and downstream. From the perspective of semiconductor production processes and equipment, ASML’s (ASML-C) lithography equipment, Canon’s semiconductor oxide coating technology, LAM’s (LRES-C) conductive layer preparation technology, and Tokyo Electron’s (TKEL-C) plasma etching technology represent oligopoly technologies in this field. From an industry perspective, these are primarily involved in manufacturing and processing semiconductor processes and equipment. In the upstream segment, the constraining technologies mainly include Wacker’s (WACK-C) polysiloxane adhesive for semiconductor sealing, Nitto Denko’s (NITL-C) polytetrafluoroethylene copolymer for semiconductor sealing and corrosion resistance, and reverse osmosis membrane technology used in ultra-purification processes for semiconductor materials. In the domain of production and inspection instruments, the key technologies encompass Zeiss’s (ZEIS-C) roughness measurement instruments, Hitachi’s radiation property testing instruments, and KLA Tencor’s (KLAT-C) wafer chip material failure detection technology. Regarding the main semiconductor products, it can be observed that processor chips are mainly controlled by Intel (ITLL-C) and Qualcomm(QUAL-C), especially in the domain of processor technology for portable devices, where Qualcomm holds overwhelming dominance with TSA and TSS values exceeding 0.8, indicating strong monopolistic advantages. On the other hand, memory chip technology is shared among four memory chip enterprises, including MICRON. Furthermore, in this area, core intermediary technologies mainly pertain to process equipment technologies, namely ASML’s lithography equipment, as well as LAM and Tokyo Electron’s equipment for semiconductor processes, such as sputtering, chemical vapor deposition, and plasma technologies. Overall, the electronic manufacturing industry exhibits a relatively high degree of monopoly characteristics. In the field of processor chips, Qualcomm and Intel hold significant monopolistic positions in their respective domains, while memory chip technology is highly competitive, with 4 enterprises owning the core technology and simultaneously competing. In terms of production equipment, the key enterprises include ASML, Tokyo Electron, and LAM. ASML, in particular, dominates the lithography sector, holding nearly 70% of the global market share in lithography machines. Similarly, KLA Tencor’s wafer and chip online inspection technology is almost required in all production lines, and recent attempts at acquisitions and mergers related to the enterprise have been prohibited by U.S. Department of Justice under antitrust laws, highlighting its dominant position. In the realm of semiconductor materials, core technologies primarily revolve around silicon surface treatment techniques and synthetic materials applied in the electronic manufacturing industry. The technologies constraining downstream products in the industry are mainly the processor chip and memory chip technologies identified through the above analysis.

**Table 5:** List of bottleneck technologies and enterprises in the electronic manufacturing industry.

No.	Technology field	Enterprise	Technology	Corresponding technology codes	Mediating technologies	Enterprise	Corresponding technology code
1	Semiconductor materials, production, processes	ASML(ASML-C)	Lithography equipment	U11-C04E1	-	-	-
		LAM(LRES-C)	Preparation of conductive layers	U11-C05C6			
		Tokyo Electron(TKEL-C)	Plasma etching equipment	U11-C09C			
		Canon(CANO-C)	Semiconductor oxide coating technology	L04-C12A			
2	Polymers, catalyst equipment, processes	-	-	-	Aggregated adhesive	Wacker(WACK-C)	G03-B02E
					Silicon surface active agents entegris	Entegris(ENTE-C)	E10-A22G
					Cyclohexane process equipment, fiber production wacker	Wacker(WACK-C)	E11-A01, A12-S05L
					Polytetrafluoroethylene homopolymer, reverse osmosis membrane	Nitto Denko(NITL-C)	A04-E08, A12-W11A
3	Semiconductor material testing, circuit testing	-	-	-	Surface roughness measurement equipment	Zeiss(ZEIS-C)	S02-A10B
					Radiation-based property detection equipment	Hitachi(HITA-C)	S02-A05A, S03-E06B1, V05-F01A1B
					Wafer chip fault detection	KLA Tencor(KLAT-C)	S01-G02B1
4	Data processing systems, internet information transfer, electronic devices qualcomm	Qualcomm(QUAL-C)	Portable device processors, switches	W01-C01D1A, W02-K07C	Lithography equipment	ASML(ASML-C)	U11-C04E1, U11-C01J2
		Intel(ITLL-C)	Data processing program control device	T01-F06	Semiconductor process sputtering, chemical vapor deposition, plasma equipment	LAM(LRES-C), Tokyo Electron(TKEL-C)	U11-C09X
		MICRON(MCRN-C), Sk-Hynix(HYNX-C)	Memory chips	T01-H01B3D			

### 4.2 Evolution characteristics of bottleneck technologies

#### 4.2.1 Overall analysis

The technologies was classified in Table 6, calculated SPC values using Pajek software, and obtained the key paths in the main route. Based on these key paths, the number of nodes and IPC code categories for each bottleneck technology’s development process were determined, as shown in Table 6. From the results below, the observation is illustrated as follows: 1) The number of nodes (<50) and IPC code categories (<20) in the fields of plasma etching equipment, semiconductor oxide coating technology, aggregated adhesives, cyclohexane process equipment, fiber production, radiation-based property detection equipment, portable device processors, and switches is relatively low. This indicates that the technology development process is either short or evolves rapidly, requiring a lower variety of technologies; 2) The number of nodes (<200) and IPC code categories (<50) in the fields of lithography light sources, silicon surface active agents, and surface roughness measurement equipment are in the middle range. This suggests that these technologies have a longer and faster development process compared to the first group; 3) In the fields of lithography equipment, wafer chip fault detection, data processing program control devices, and memory chips, the number of nodes is relatively high (>200), but IPC code categories are not as numerous (<50). This implies a higher level of technology focus and a narrower range of application, but a faster rate of technological advancement; 4) In the fields of conductive layer preparation, polytetrafluoroethylene homopolymers, and reverse osmosis membranes, both the number of nodes and IPC code categories are relatively high (>200 and >50, respectively). This indicates that these technologies evolve rapidly, have a wide range of sources, and to certain extent, possess characteristics of disruptive technologies.

**Table 6:** Number of nodes and IPC codes categories in key path of bottleneck technologies.

Subdivision	Technology field	Enterprise	Technology code	Patent amount of key route network	Type amount of Patent IPC
Semiconductor materials, production, processes	Lithography equipment	ASML	U11-C04E1	208	22
	Preparation of conductive layers	LAM	U11-C05C6	216	52
	Plasma etching equipment	Tokyo Electron	U11-C09C	36	14
	Semiconductor oxide coating technology	Canon	L04-C12A	38	6
Polymers, catalyst equipment, processes	Aggregation adhesives, ring processes, fiber production	Wacker	G03-B02E, E11-A01, A12-S05L	24	15
	Silicon surface active agents entegris	Entegris	E10-A22G	111	33
	Polytetrafluoroethylene homopolymer, reverse osmosis membrane	Nitto Denko	A04-E08, A12-W11A	216	61
Semiconductor material testing, circuit testing	Surface roughness measurement equipment	Zeiss	S02-A10B	115	38
	Radiative property testing instruments	Hitachi	S02-A05A, S03-E06B1, V05-F01A1B	48	15
	Wafer chip fault detection	KLA Tencor	S01-G02B1	309	19
Terminal product components and programs	Portable device processors, switches	Qualcomm	W01-C01D1A, W02-K07C	46	14
	Data processing program control device	Intel	T01-F06	216	38
	Memory chips	MICRON, Samsung, Sk-Hynix	T01-H01B3D	360	22

#### 4.2.2 Technical trend analysis

**Fig 6:**
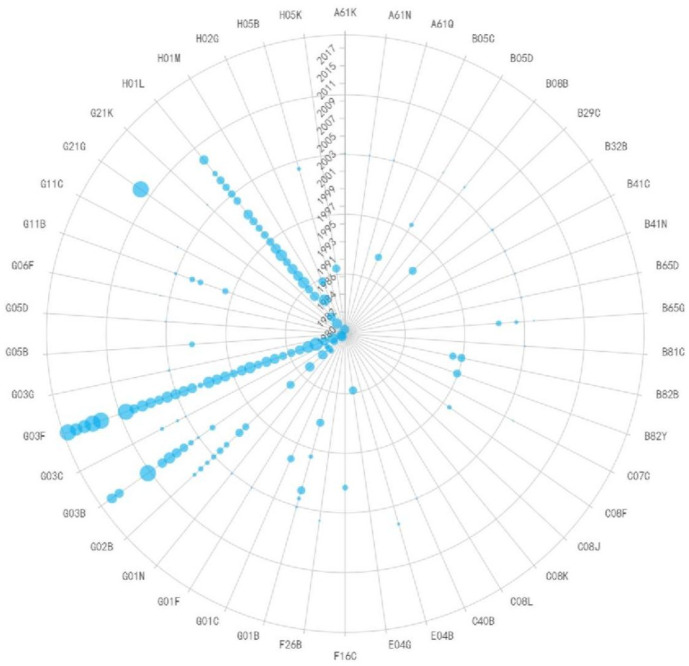
Evolutionary trend of IPC codes in lithography equipment technology.

G03F is the most commonly used IPC code in lithography equipment technology, primarily representing the photolithographic patterning process, as shown in Fig 6. In the early stages of technology development, the focus was mainly on device manufacturing and process improvement. Before 2003, the primary IPC codes were H01B+G02B or G01B or G01C, indicating that the technology at that time was primarily used for X-ray lithography mask and its manufacturing methods, as well as mask manufacturing methods and light source calibration methods using masks. After 2007, G03B replaced G02B as the main IPC code, indicating that patents during this period mainly related to improvements in refractive projection lenses. From the patent nodes during this stage, technological improvements were primarily focused on immersion lithography refractive projection lenses. In recent years, various compounds have appeared prominently in patents, indicating a current focus on the development of lithography materials, primarily related to EUV lithography machine photoresist materials, such as photosensitive amplifying anti-etching materials and photoacid generators, which have become hot topics in recent research and development. Photoresist is a photosensitive material and selective resistance etching material used to achieve pattern transfer in lithography processes. In lithography, it is used to selectively shield or protect the substrate surface from etching. It can be broadly categorized into materials sensitive to ultraviolet light, deep ultraviolet light, EUV light, X-rays, and materials sensitive to high-energy particle beams such as electron and ion beams. With the continuous improvement of lithography performance, there is a need to develop materials with higher resolution, making it a hot research area.

**Fig 7:**
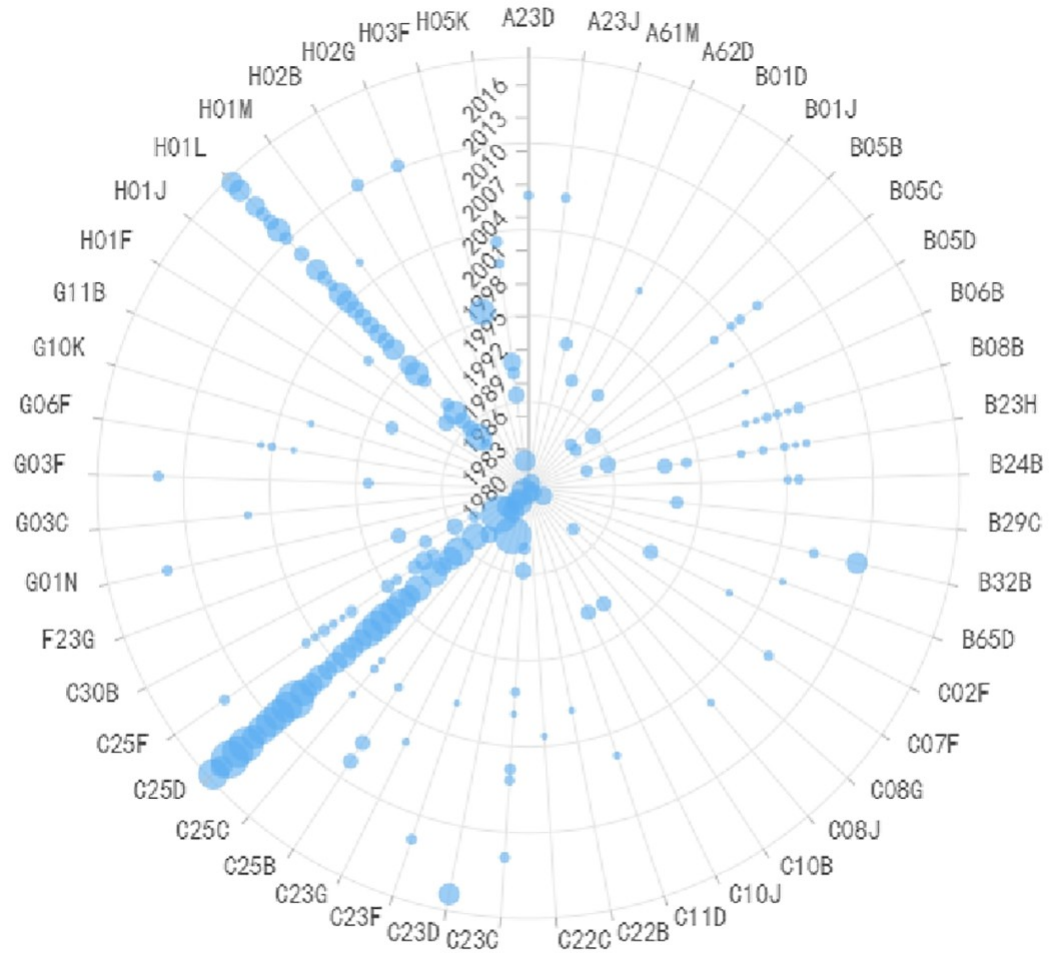
Evolutionary trend of IPC codes in preparation of conductive layers equipment.

**Fig 8:**
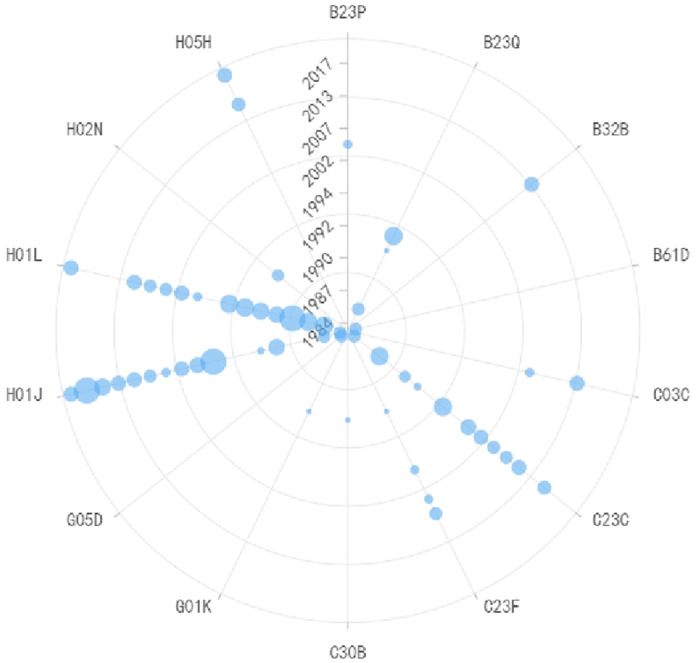
Evolutionary trend of IPC codes in preparation of plasma etching equipment.

Due to the strong correlation between the preparation of conductive layers and plasma etching equipment, as shown in Figs 7 and 8 respectively, the paper discusses these two technologies together. C25D (Methods for Electrolytic or Electrophoretic Production of Coatings) and H01L (Methods or Devices Specifically Ddapted For Manufacturing or Processing Semiconductor or Solid-State Devices or Their Components) are the most crucial IPC codes in the preparation of conductive layers in semiconductor production equipment, representing all processes related to thin film deposition and plasma etching. From a temporal perspective, before 2007, IPC codes were primarily located on the right side of the graph, including A61M, B05C, B08B, B23H, B23Q, etc. These primarily involved equipment coating, cleaning, and processing techniques, as well as related measurement and detection technologies. Subsequently, dry etching technology gradually replaced wet etching technology, and electrochemistry played a more important role. IPC codes gradually shifted to the left side of the graph, including C23C (Plating on Metal Materials), C25F (Methods for Removing Materials from Objects by Electrolysis), and G01N (Testing or Analyzing Materials by Determining Their Chemical or Physical Properties). These focused on improving equipment precision through electrochemical methods. During this period, the emergence of H05H (Plasma Technology) was significant, and plasma etching technology became mainstream in the industry. In the future, etching processes become increasingly precise, and Atomic Layer Etching (ALE) equipment will be the next-generation mainstream etching equipment. Considering the continuous reduction in critical dimensions of chips and the emergence of three-dimensional (3D) structures like Fin Field-Effect Transistor (FinFET) and 3DNAND, plasma etching faces challenges such as differences in etching rates and damage to underlying materials. ALE can selectively remove target materials at the atomic scale without damaging other parts of the structure. It can etch grooves with gaps at the atomic width level. Its precision control of removed material quantity without affecting the properties of other parts is suitable for directional etching. ALE can be divided into Plasma ALE and High-Temperature ALE. Currently, Plasma ALE has entered the production phase, while High-Temperature ALE is still in its early stages and has a long way to go before commercial use. Overall, although ALE has not yet replaced traditional plasma etching processes in the semiconductor manufacturing field, it is used for precise removal of materials at the atomic level. The industry is committed to developing the next generation of ALE technology for advanced logic processors and memory production. Chip manufacturers are also exploring all possibilities of ALE. With the development of 3D technology and the limitations of traditional etching processes, ALE equipment is expected to become the next-generation mainstream etching equipment, experiencing rapid growth.

**Fig 9:**
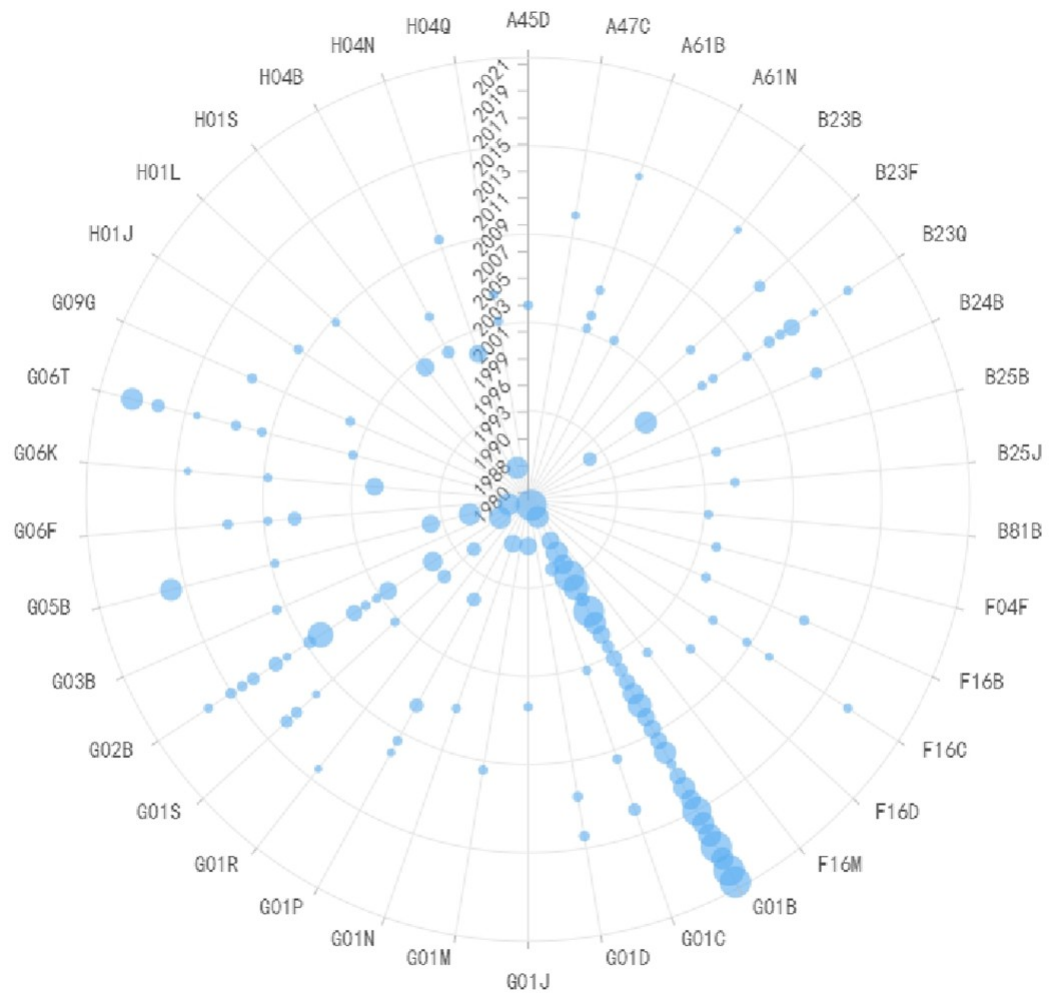
Evolutionary trend of IPC codes in roughness measurement technology.

**Fig 10:**
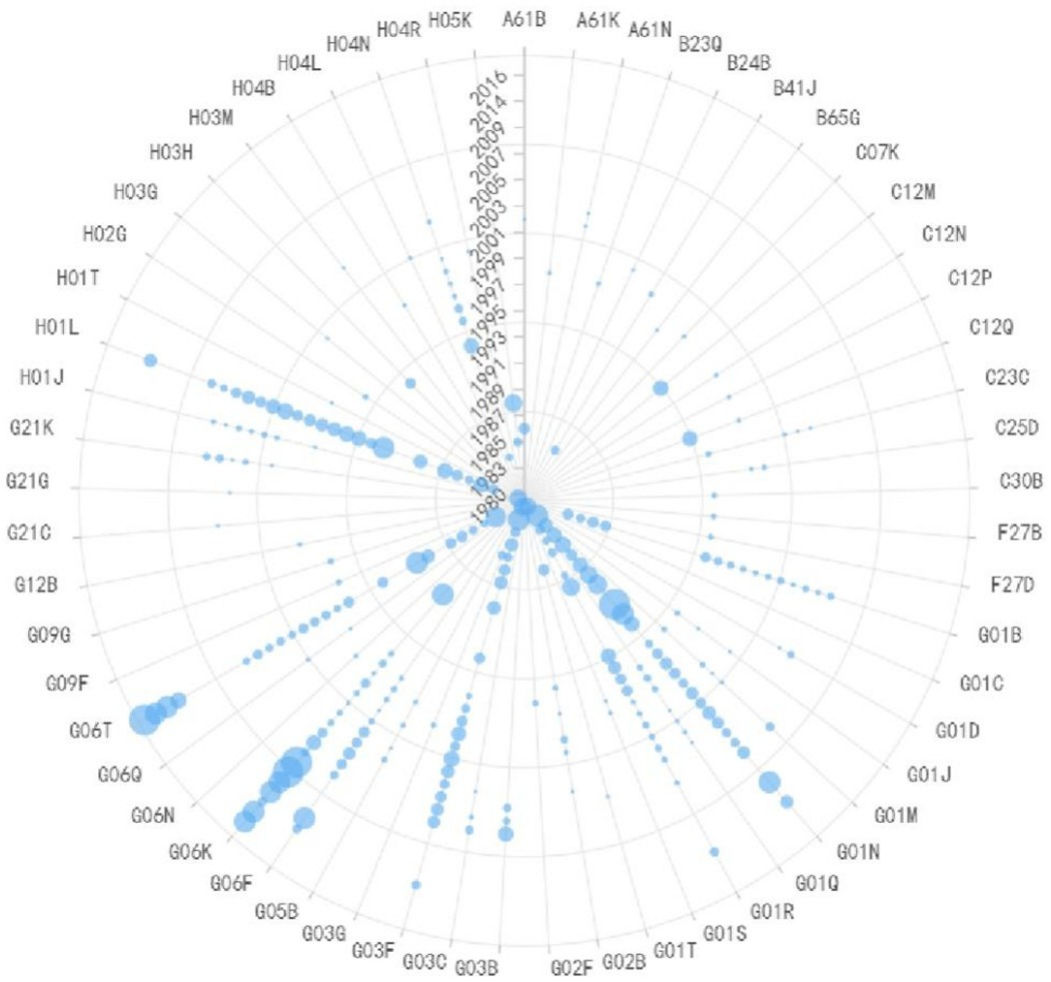
Evolutionary trend of IPC codes in fault detection of wafer chips technology.

Roughness measurement and wafer chip fault detection are closely related, as shown in Figs 9 and 10 respectively, and therefore, this paper discusses them together. For roughness measurement technology, G01B (Measuring Lengths, Thicknesses, or Similar Linear Dimensions) is the generic IPC code in this field, and it has remained constant throughout the technological development process. However, other codes have evolved over time. Before 2015, G02B (Optical Elements, Systems, or Apparatus) and B23Q (Parts, Components, or Accessories for Machine Tools, such as Copying Devices or Control Devices) were the primary IPC codes, indicating that roughness measurement was predominantly based on optical principles during that period. Common non-contact measurement instruments included white light interferometers and laser confocal microscopes. Starting from 2016, G06T (General Image Data Processing or Generation of Subcategories for General Image Data Processing) and G05B (General Control or Regulating Systems) began to take a prominent position, signifying that image-based measurement techniques gained significance during this period. For instance, Atomic Force Microscopy (AFM), a representative instrument in the field of scanning probe microscopy, gained widespread application in the electronic manufacturing industry. AFM measures surface roughness by detecting changes in the bending of the AFM probe cantilever caused by the interaction forces between the probe and the sample surface. In terms of detection technology, the developments post-2014 mainly revolved around G06T (General Image Data Processing or Generation of Subcategories for General Image Data Processing) and G06K (Graphic Data Reading). This was mainly driven by the requirements of the semiconductor manufacturing process. Various image technologies are needed for tasks such as mask defect localization and verification, zero-defect repair, mask fine-tuning, and mask measurement. For instance, repairing objects may include various shaped defects on binary masks, phase-shift masks, and EUV masks, which requires multiple exposure techniques and complex layer splitting. In order to carry out these high-precision tasks, measurement systems require advanced image measurement technology and more sophisticated deep learning algorithms. Therefore, the future direction is likely to focus on highly-resolved digital and intelligent solutions to meet these evolving needs.

**Fig 11:**
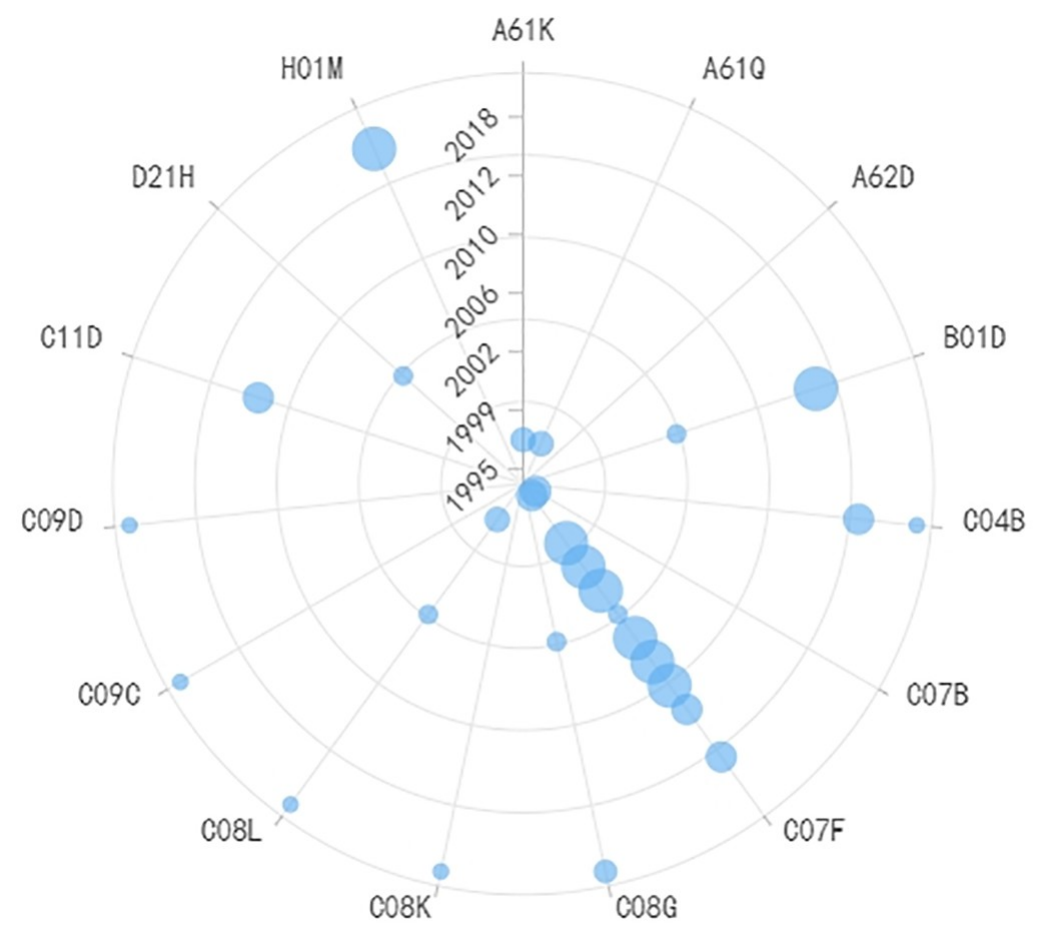
Evolutionary trend of IPC codes in materials of Wacker.

**Fig 12:**
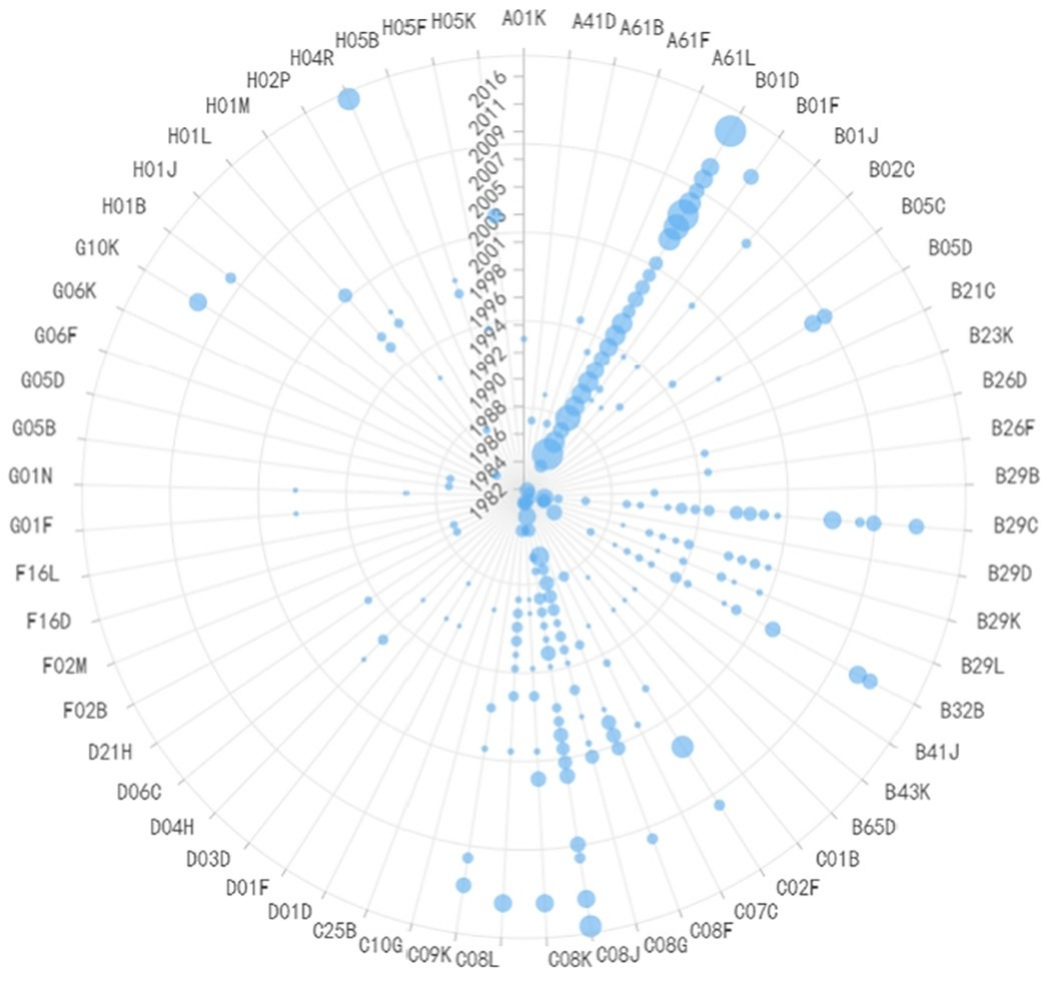
Evolutionary trend of IPC codes in materials of Nitto Denko.

**Fig 13:**
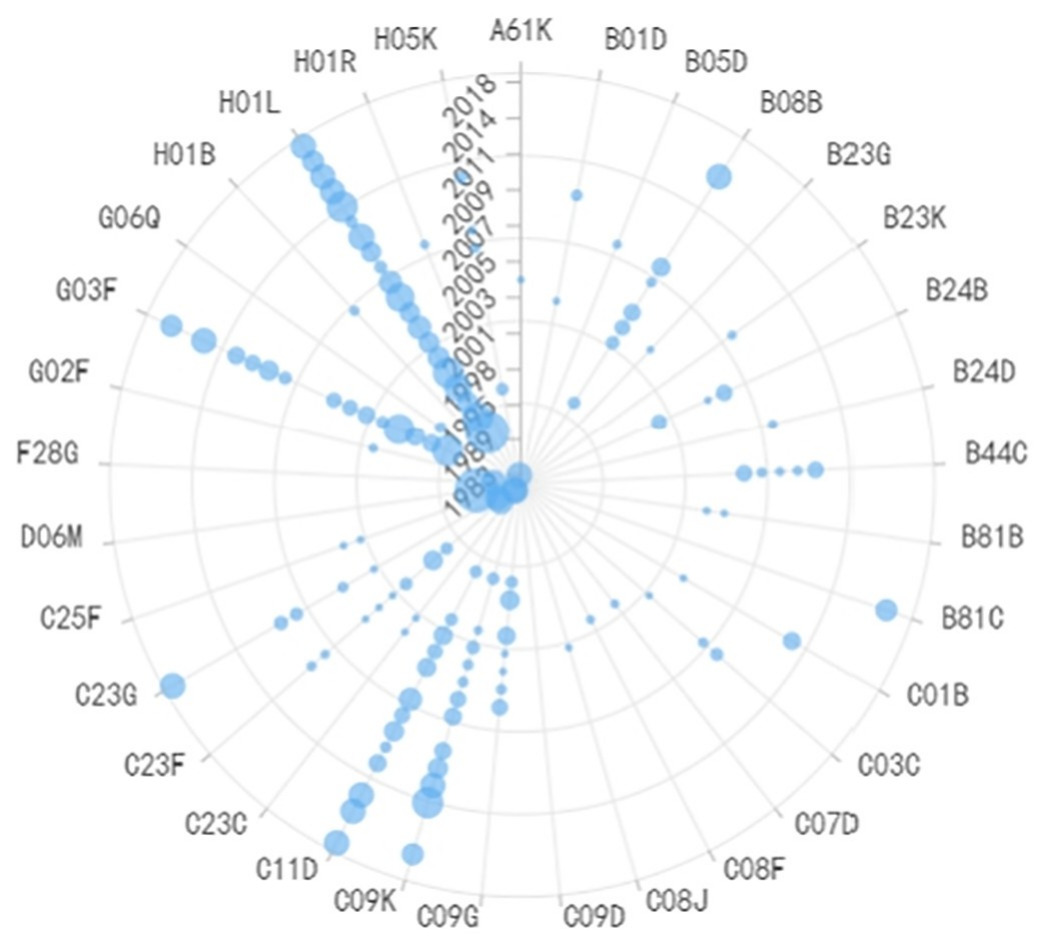
Evolutionary trend of IPC codes in materials from of Entegris.

In the field of materials, bottleneck technologies tend to have a relatively slower pace of knowledge updates compared to other domains. For instance, in the case of Wacker’s condensation adhesive, ring-closure process equipment, and fiber production processes, there have been only 7 significant milestones in their development, occurring in 1995, 1999, 2002, 2006, 2010, 2012, and 2018, as shown in Fig 11. On average, there is a 3.8-year gap between each set of important patents. Entegris and Nitto Denko exhibit similar trends, as shown in Figs 12 and 13 respectively, with Entegris having a 3.2-year gap and Nitto Denko a 2.3-year gap between significant developments. For Entegris’ silicon surface-active agents, G03F (Lithography for Patterned Surfaces), H01L (Semiconductor Devices), C11D (Detergent Compositions), and C09K (Various Applications Materials in Other Classes) serve as the fundamental technologies, and therefore, they appear in the foundational patents throughout the years. In recent years, codes related to cleaning methods like B08B (Methods Using Liquids or Vapors for Cleaning) and B81C (Apparatus or Systems for Manufacturing or Treating Substrates) have gradually emerged. Silicon wafer production processes involve mechanical grinding steps, which utilize chemical grinding fluid formulations to remove excess conductive or insulating materials from the surface of integrated devices, achieving a flat, smooth surface for developing multilayer integrated circuits. In the cleaning step, removing nanometer-sized particles, reducing potential wafer defects, and maintaining the integrity of already placed material layers are crucial aspects of the process. With the advent of processes at 10 nm and below, there has been a change in the quantity and type of films and materials that come into contact with the cleaning process, leading to inevitable updates in cleaning processes and products. For Nitto Denko, B01D (General Physical or Chemical Methods or Apparatus) and C08J (Processing of Organic Polymeric Compounds) serve as the foundation for their technology applications, indicating the predominant use of organic chemical substances as cleaning agents. The emergence of IPC code G10K (Sound-Producing Devices) in recent years suggests that Nitto Denko has gradually shifted from steam cleaning to ultrasonic cleaning methods.

**Fig 14:**
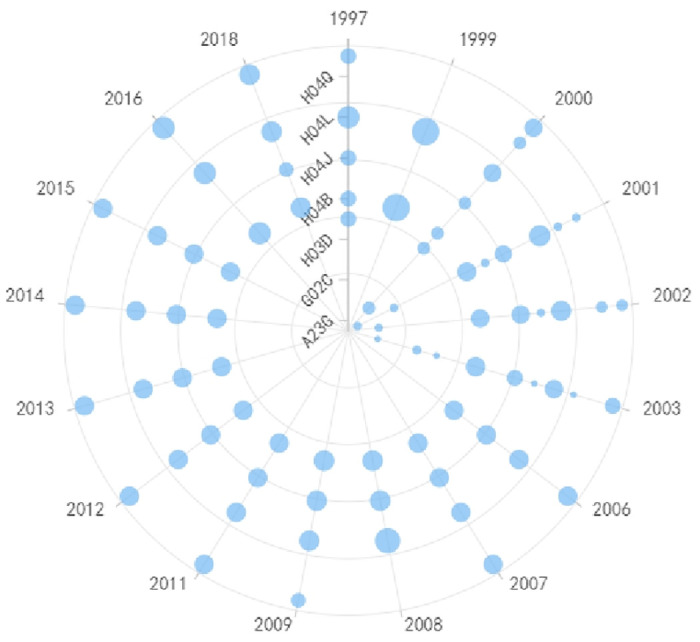
Evolutionary trend of IPC codes in chip for mobile communication devices.

**Fig 15:**
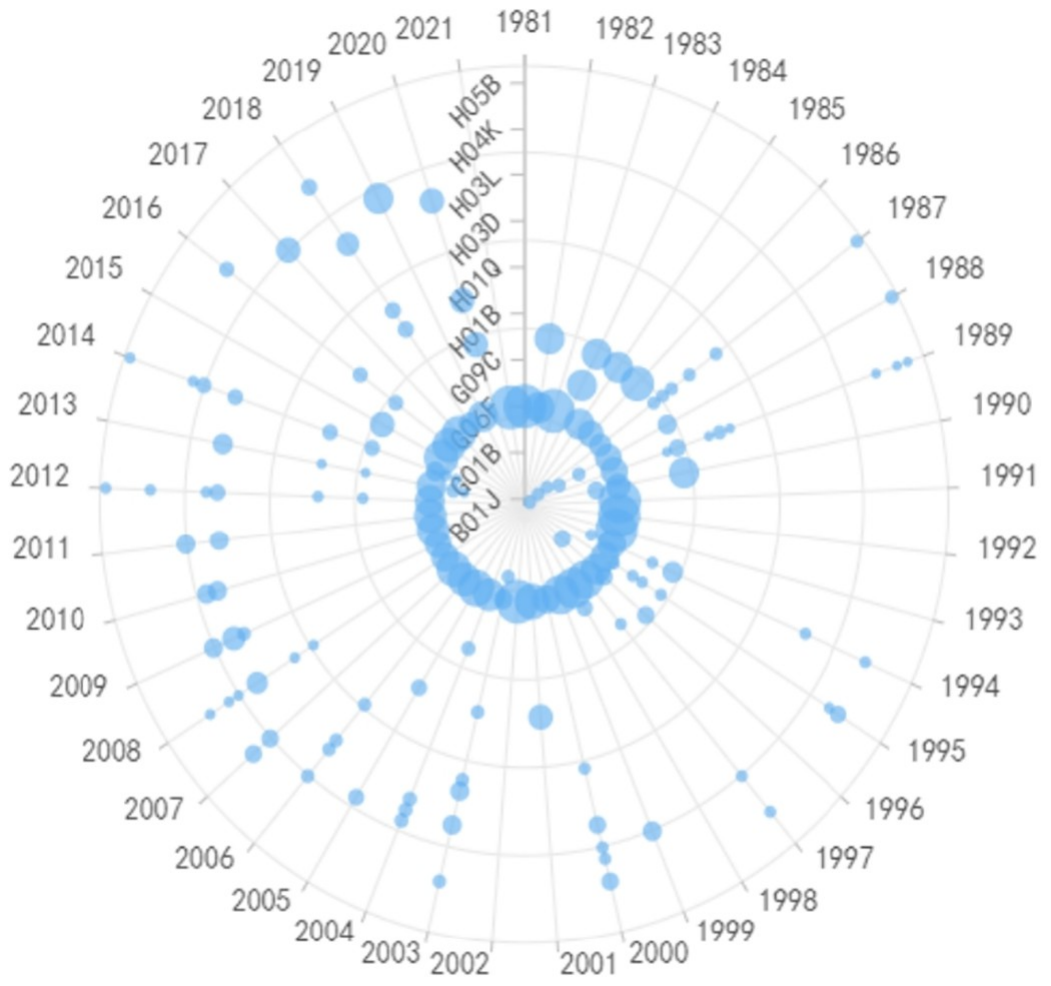
Evolutionary trend of IPC codes in a computer chip.

**Fig 16:**
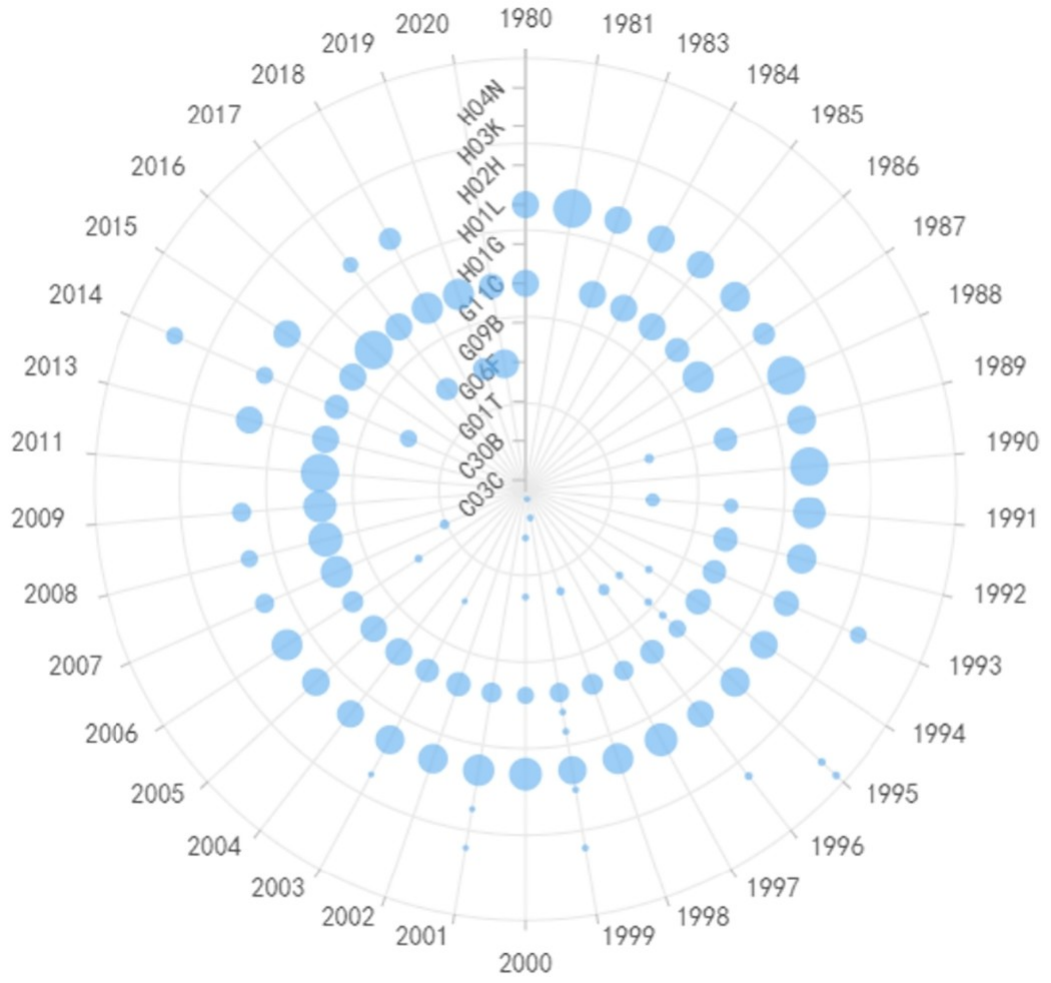
Evolutionary trend of IPC codes in a memory chip.

The bottleneck technologies in terminal product components and programs primarily involve various algorithmic and related chip technologies. For instance, W01-C01D1A is primarily associated with chipsets for mobile communication devices, as shown in Fig 14, T01-F06 mainly pertains to computer chips as shown in Fig 15, and T01-H01B3D primarily relates to memory chips as shown in Fig 16. When examining the characteristics of IPC code changes, except for a notable change in computer chips around 2000, most of them have remained stable, indicating that the technology fields used in these chips have not seen significant updates. Moreover, compared to other technologies, these three technologies exhibit faster update rates, with significant milestones (patents) almost every year. Although various algorithms are the development focus in chip technologies, fundamentally, semiconductor process miniaturization is an essential foundation for these technologies. This is because various chip architectures and algorithms are designed for processing larger-scale data, which inherently requires higher chip manufacturing processes. Since 2018, FinFET technology, which has been leading semiconductor chip manufacturing processes, has been losing ground. The main reason is that the wafer nodes in chip manufacturing have reached the stage of mass production at 10nm, which is very close to the physical limit of FinFET technology at 5 nm. The cost of advancing beyond this point has become increasingly prohibitive. As a result, Gate-All-Around Field-Effect Transistor (GAAFET) technology is on the rise. In the future, technologies such as artificial intelligence and 6G require handling significantly larger amounts of data. To process such vast data quantities, even more precise 3 nm manufacturing processes are needed compared to the current 5nm manufacturing process.

## 5 Discussion

This study identified enterprises in the electronic manufacturing industry and related sectors from the Orbis database. Subsequently, it utilized the technical codes extracted from the patent data of these enterprises to establish a social network, pinpoint key technical codes, and compute the technical share metrics associated with these key codes. A limited number of enterprises have achieved proficiency in certain key technologies, thereby pinpointing bottleneck technologies. Subsequently, the research focus transitioned towards strategies for overcoming these technologies. The research employed Pajek software to analyze the developmental trajectories of different bottleneck technologies. It aimed to outline their distinctive features, identify technologies that are relatively easier to overcome, pinpoint those necessitating extensive technological groundwork, recognize technologies with disruptive attributes, and propose novel approaches for further exploration.

Firstly, by leveraging the information and patent databases of Orbis company, the bottleneck technologies was identified within the industry. According to pertinent news sources [34–36], the technologies that have been identified may be considered bottleneck technologies, frequently under the control of monopolistic enterprises. Therefore, it is suggested that this approach still retains a degree of efficacy. By utilizing the Obis company information database, this study aims to establish a comprehensive industry chain, encompassing the primary stakeholders within the industry. This information is of paramount importance for policymakers, as the majority of them lack prior knowledge of the industry chain’s composition. Frequently, conducting enterprise research or identification is necessary within a limited scope.

Secondly, semiconductor chips represent only one of the bottleneck technologies within the electronic manufacturing sector. Bottlenecks are evident in multiple facets of the electronic manufacturing sector, encompassing auxiliary materials, processing equipment, testing technologies, and more. The prevalence of bottleneck technology is pervasive throughout the industry, with semiconductor chips standing out as a critical bottleneck component in the final product.

Finally, the study involved an examination and calculation of the development trajectory of bottleneck technology. It also included the decomposition of the functional composition and evolution of each technology using patent citation data. In contrast to prior research that has traditionally utilized the quantity of patents as a criterion for identifying breakthrough points [37], the approach of functional evolution may offer greater operational benefits.

## 6. Conclusion and Implications

### 6.1 Conclusion

Despite the significant attention bottleneck technology has garnered from various levels of Chinese government, the journey towards resolving it has been fraught with challenges. Bottleneck technology extends beyond chips and is prevalent in various stages of the industry, including upstream, midstream, and downstream processes. The upstream sector of an industry typically serves as the cornerstone, thus holding greater significance. The research results indicate the presence of bottleneck technology in the process equipment, materials, and key components. Therefore, developments in these technologies cannot be instantaneously attained and require a gradual approach. Meanwhile, the electronic manufacturing industry represents only a fraction of the broader landscape, indicating the necessity for further exploration of bottleneck technologies in various other sectors.

It is believed that within the realm of electronic manufacturing, China should strive for advancements in two specific areas. The initial direction encompasses a range of technologies, such as plasma etching equipment, semiconductor oxide coating technology, polymer adhesives, closed-loop process equipment, fiber production, radiation material performance testing instruments, and other related technologies. The development cycle of these technologies is relatively short, leading to frequent updates and requiring a reduced variety of technologies. Therefore, breaking through these technologies is relatively simple. The second direction encompasses technologies associated with the fabrication of conductive layers, polytetrafluoroethylene polymers, and reverse osmosis membranes. These technologies originate from diverse sources initially and demonstrate distinct characteristics of updates and substitutions. China has the potential to promptly identify competitive new opportunities in these sectors.

China should persist in investing in and advancing lithography equipment, wafer fault detection, and chip technology for sustained growth and development in the long run. These technologies necessitate a heightened level of specialization and more profound technical expertise, with their technological development progressing relatively swiftly. Therefore, it is imperative to emphasize sustained and stable investment in research and development to bridge the gap with other prominent nations.

### 6.2 Implications

This study primarily holds two significant implications for management. This paper proposes a method for the identification and selection of bottleneck technologies by leveraging data from multiple sources. This approach does not depend on expert interviews or research methodologies commonly employed in existing literature. Instead, it utilizes widely-used commercial databases to promptly pinpoint bottleneck technologies within the specified field. The selection of the target field range can be tailored based on industry categories, offering control and adjustability. Therefore, conducting bottleneck technology early warning in various fields is of great significance. Additionally, the paper aims to employ the primary developmental pathways of patent citation analysis technology to comprehend its evolutionary traits. In contrast to conventional main path analysis, the investigation examined the technical function codes along the path, offering a more intricate delineation of the pathway. Therefore, it is also crucial for research management personnel to develop and structure research plans.

The electronic manufacturing industry holds significant practical importance as it plays a pivotal role in the economic development of the country and serves as a focal point for technological innovation. In 2018, the focal point of the trade dispute between China and the United States revolved around ZTE Corporation within the electronic manufacturing sector, permeating the broader landscape of technological competition between the two nations. For China, it is important to initiate research at an early stage to develop an early warning mechanism. Therefore, the systematic identification of bottleneck technologies is of particular significance.

### 6.3 Deficiencies and future prospects

In terms of results, the method used in this study has shown certain utility in identifying bottleneck technologies. However, it is important to note that the accuracy of the results depends on the completeness of the industry structure. If some core enterprises are missing from the analysis, it could introduce errors into the results. Additionally, in this research, the assessment of bottleneck technologies is based on a comparison of patent dominance. In other words, this method may not be effective in industries with fewer patent applications or industries that do not heavily rely on patent protection. For instance, in many traditional metalworking industries, certain enterprises play crucial roles in the industry, but their innovations may not be reflected through patents. For example, there are numerous hidden champions in Germany’s metal fastener industry, and these industries often have irreplaceable roles in aviation and high-speed railways. These are the kinds of issues that will need further discussion in future research.

## Supporting information

S1 Table Table of correlation coefficient matrix of enterprise patent code. (XLSX)
